# The Origin, Succession, and Predicted Metabolism of Bacterial Communities Associated with Leaf Decomposition

**DOI:** 10.1128/mBio.01703-19

**Published:** 2019-09-03

**Authors:** Sara L. Jackrel, Jack A. Gilbert, J. Timothy Wootton

**Affiliations:** a Department of Ecology & Evolution, University of Chicago, Chicago, Illinois, USA; b Scripps Institution of Oceanography, University of California San Diego, La Jolla, California, USA; University of California, Irvine

**Keywords:** bacterial metabolism, aquatic decomposition, ecosystem subsidies, intraspecific variation, environmental filtering, plant defensive chemistry, bacterial metabolism

## Abstract

Intraspecific variation in plant nutrient and defensive traits can regulate ecosystem-level processes, such as decomposition and transformation of plant carbon and nutrients. Understanding the regulatory mechanisms of ecosystem functions at local scales may facilitate predictions of the resistance and resilience of these functions to change. We evaluated how riverine bacterial community assembly and predicted gene content corresponded to decomposition rates of green leaf inputs from red alder trees into rivers of Washington State, USA. Previously, we documented accelerated decomposition rates for leaves originating from trees growing adjacent to the site of decomposition versus more distant locales, suggesting that microbes have a “home-field advantage” in decomposing local leaves. Here, we identified repeatable stages of bacterial succession, each defined by dominant taxa with predicted gene content associated with metabolic pathways relevant to the leaf characteristics and course of decomposition. “Home” leaves contained bacterial communities with distinct functional capacities to degrade aromatic compounds. Given known spatial variation of alder aromatics, this finding helps explain locally accelerated decomposition. Bacterial decomposer communities adjust to intraspecific variation in leaves at spatial scales of less than a kilometer, providing a mechanism for rapid response to changes in resources such as range shifts among plant genotypes. Such rapid responses among bacterial communities in turn may maintain high rates of carbon and nutrient cycling through aquatic ecosystems.

## Abstract (precis)

Community ecologists have traditionally treated individuals within a species as uniform, with individual-level biodiversity rarely considered as a regulator of community and ecosystem function. In our study system, we have documented clear evidence of within-species variation causing local ecosystem adaptation to fluxes across ecosystem boundaries. In this striking pattern of a “home-field advantage,” leaves from individual trees tend to decompose most rapidly when immediately adjacent to their parent tree. Here, we merge community ecology experiments with microbiome approaches to describe how bacterial communities adjust to within-species variation in leaves over spatial scales of less than a kilometer. The results show that bacterial community compositional changes facilitate rapid ecosystem responses to environmental change, effectively maintaining high rates of carbon and nutrient cycling through ecosystems.

## INTRODUCTION

Millions of years of coevolution between plants and their herbivores and pathogens have in part caused the diversity of plants we see today (1, 2). Plant chemical and mechanical defenses have played a critical role in this diversification, with over 200,000 described plant secondary metabolites that range from mild to high toxicity with an array of chemical structures with varied modes of action (3–5). Just as herbivores and pathogens have spurred plants to biosynthesize this diverse arsenal, bacteria have evolved to degrade these toxins. These bacteria, which may be either free-living decomposers of plants or symbiotic with an herbivore, have evolved diverse enzymatic pathways to degrade plant toxins with the benefit of unlocking this energy source (6). Bacterial detoxification and consumption of plant detritus are essential for maintaining the availability of carbon and nutrients for nonbacterial organisms within food webs (7).

While plants, herbivores, and bacteria have coevolved over long periods, the interactions among them are continually in flux. Evolution can occur over directly observable, ecological time frames, which has opened new questions about the roles of ecological versus evolutionary mechanisms in regulating ecosystem-level function (8). Bacteria in particular have short generation times. This could facilitate their rapid adjustment in the face of changing environmental conditions, such as changes in the distribution of plant resources, via either evolutionary or ecological mechanisms, such as environmental filtering.

In this study, we specifically evaluate the role of ecological mechanisms by investigating whether bacterial diversity, community composition, and gene content associated with metabolic pathways to breakdown plant secondary metabolites correspond to patterns of locally accelerated decomposition in a riverine system (9–11). Locally accelerated decomposition, or the “home-field advantage,” in which decomposers more rapidly decompose leaves from trees of local origin, was originally documented by Gholz et al. (9). Further studies have shown the home-field advantage may be a widespread phenomenon, particularly in the soils of mature forests protected from most anthropogenic disturbances, where 77% of 35 studies demonstrated locally accelerated decomposition, with an average of 8% faster decomposition at Home locations (12, 13). More recently, the home-field advantage has been documented in aquatic systems where terrestrial leaf litter falls into rivers (14, 15). Further, this pattern has more recently been shown to also occur over small geographical scales of less than 1 km to individual trees within the same species (11). This local preference among aquatic and soil decomposers is driven in part by geographically variable secondary metabolites that are produced by plants to deter feeding by terrestrial herbivores (10). Such patterns of local acceleration could have arisen from either ecological or evolutionary mechanisms, including shifts in decomposer community composition, plasticity within decomposer individuals, or genetic change among populations of decomposers. To begin to unravel the underlying drivers of locally accelerated decomposition, we evaluate ecological mechanisms within bacteria.

We evaluate the role of bacteria as one component of the larger aquatic decomposer assemblage that includes fungi and invertebrates. Studies have found that leaf mass loss in stream systems is the consequence largely of invertebrates, followed by fungi and bacteria (16). However, taxonomic and functional databases of bacteria have been more thoroughly developed than those of fungi. Given the analytical tools currently available, we chose to focus on aquatic bacteria but note that fungi are key players in this ecosystem function. Additionally, although aquatic macroinvertebrates are important consumers of plant detritus in streams (17, 18), we previously found no evidence that aquatic invertebrates drove patterns of locally accelerated decomposition in streams because their abundance, taxonomic diversity, and functional diversity were similar among leaf packs of local versus nonlocal origin (14). A possibility that we do not investigate in this study is whether variation in aquatic macroinvertebrate gut microbiomes may drive locally accelerated leaf decomposition. We focus our study on the role of aquatic bacterial communities in driving locally accelerated leaf decomposition because of (i) the similar compositions of aquatic invertebrates despite differences in leaf decomposition; (ii) the ability of microbes to grow more rapidly, which would facilitate more fine-scale shifts in community composition in adjustment to plant traits; and (iii) the capacity of bacteria to detoxify plant secondary metabolites.

Here, we document the occurrence and successional patterns of free-living bacteria on red alder leaves derived from trees of local and nonlocal origin in a reciprocal green leaf pack experiment completed in streams. We test our hypothesis that leaves of local origin will harbor a more specialized community of aquatic bacteria over the course of decomposition that is better able to rapidly break down the abundant local food source. Second, we test our hypothesis that the aquatic bacterial community inhabiting local leaves will undergo more rapid successional change than communities inhabiting leaves of nonlocal origin. For example, communities inhabiting local leaves might be more efficient at degrading secondary metabolites typical of a local site and therefore shift more rapidly to a community dominated by generalists that break down plant polysaccharides. In contrast, communities inhabiting nonlocal leaves may require a greater relative abundance and longer residence time of those bacterial taxa that are less efficient at metabolizing locally novel plant secondary compounds. For example, a greater relative abundance might occur if the degradation of these atypical metabolites requires multiple steps completed by more than one taxon whereas degradation of typical metabolites might require only a single taxon. Further, a longer residence time might be required if bacterial taxa which are not locally adapted are less efficient at degrading atypical metabolites and therefore do so at a lower rate. Overall, by evaluating differences in aquatic bacterial community composition, we provide insight into the potential mechanisms driving locally accelerated decomposition in streams, which may ultimately elucidate the tempo and trajectory of ecological changes within aquatic bacterial communities that confer locally beneficial function. Further, unraveling the underlying mechanisms of how bacteria might be adjusting to novel combinations of plant secondary metabolites may be pertinent for the field of bioremediation.

## RESULTS

**FIG 1:**
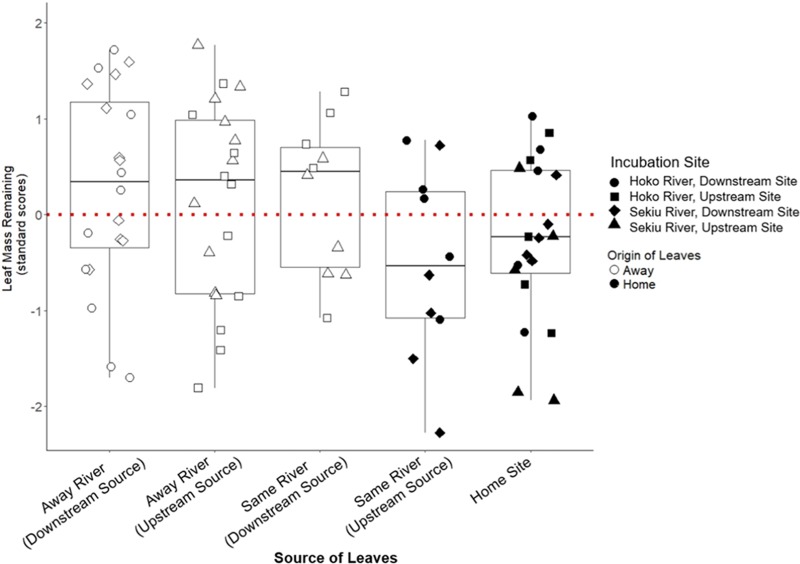
Decomposition measures of leaves from 20 riparian red alder trees used in a reciprocal transplant experiment across the Hoko and Sekiu Rivers, WA, USA. Leaves of local origin decomposed significantly faster than leaves from trees growing in locations that would not naturally reach the incubation site, including trees growing along the same river but downstream of the incubation site and trees growing at the other river. Note that the *y* axis depicts decomposition rates as standardized scores (i.e., z-scores), in which decomposition rates within an incubation site are adjusted to μ = 0, SD = 1, so a *y* axis value = 1 indicates 1 SD above the mean decomposition rate at that incubation site. For example, the standardized score for a leaf pack, referred to as “pack 1,” incubating at the Hoko River, Downstream Site would equal (% mass loss of pack 1 at Hoko, Downstream − mean % mass loss of all packs incubating at Hoko, Downstream)/(standard deviation of % mass loss of all packs incubating at Hoko, Downstream). This standardization serves to illustrate the relative decomposition rates of Home versus Away leaves at each site rather than variation among sites; however, our mixed-effects model was run on the nonstandardized decomposition data with incubation site as a random effect term (leaf origin term: *F*_4,68_ = 2.6, *r_s_P_C_* = 0.3, *P* = 0.01). For nonstandardized data, see Fig. S8. Categories on the *x* axis include, from right to left, trees growing immediately upstream of the incubation site and trees growing further upstream of that incubation site at the “Away Site” on the same river, both of which are considered “Home,” and then trees growing downstream of the incubation site at the “Away Site” on the same river, trees growing at the upstream site on the paired river, and trees growing at the downstream site on the paired river, all three of which are considered “Away.” Note that all points are horizontally jittered to minimize overplotting.

Leaves of Home origin lost significantly more leaf mass by decomposition than leaves of Away origin (Fig. 1) (analysis of variance [ANOVA] ordered-heterogeneity test, *F*_4,68_ = 1.8; *r_s_P_C_* statistic = 0.75; *P* < 0.01; see Materials and Methods for further description of the *r_s_P_C_* statistic). Home leaves were those incubating at a site immediately downstream of their parent tree (i.e., Home as shown in Fig. S1 in the supplemental material), as well as those incubating at a site further downstream of the parent tree (i.e., “same river downstream” as shown in Fig. S1). Away leaves were those incubating at a site upstream of their parent tree (i.e., “same river upstream,” as shown in Fig. S1), as well as those incubating at a different river from where their parent tree was growing (i.e., “away river” as shown in Fig. S1). This result of a significant home-field advantage was also consistent with findings using an alternative method that is more commonly used in terrestrial systems (13). For this alternative method, we used the subset of 20 leaf packs in our experiment that test for our main contrast across rivers, rather than Away sites within the same river (i.e., we used all leaf packs deployed on the “home site, on the home river” and one corresponding leaf pack on the Away river). We found Home leaves decomposed significantly more than Away leaves (*t*_9_ = 2.60, *P* = 0.029, median home-field advantage index [HFAI] = 44%; see the work of Ayres et al. [13] for details of HFAI calculation). Further, as previously reported by Jackrel et al. (10), the red alder trees used in this reciprocal transplant experiment differed significantly across the four riparian sites in their relative abundance of aromatic secondary metabolites, including ellagitannins and diarylheptanoids (see Fig. S2 for details). We therefore focus a portion of our analysis on bacterial metabolism of aromatic compounds.

10.1128/mBio.01703-19.2 FIG S1 Illustration of experimental design for the reciprocal transplant experiment. Also listed are measurements of temperature, photosynthetically active radiation (PAR), flow rate, conductivity, pH, and dissolved oxygen from July 2013 for each of the four sites used in the reciprocal transplant experiment. Measures of temperature and PAR are means over 7-day periods, except for the Sekiu upstream site (2 days). Daytime temperatures were determined from 10-min interval readings using HOBO data loggers for all nonzero light measurements. Maximum and minimum temperatures include daytime and nighttime readings. Flow rates were measured during 0.5-min increments using a Global Water flow probe. Dissolved oxygen, conductivity, and pH were measured using a Hach HQ40d multiprobe. Download FIG S1, PDF file, 0.2 MB. Copyright © 2019 Jackrel et al. 2019 Jackrel et al. This content is distributed under the terms of the Creative Commons Attribution 4.0 International license.

10.1128/mBio.01703-19.3 FIG S2 (A) Leaves of individual red alder trees growing in the riparian zones of two rivers vary in the relative abundance of 35 secondary metabolites, including ellagitannins and diarylheptanoids, by tree origin via discriminant function analysis. (B) Loadings of each chemical variable, with tentative chemical identifier, are shown in the corresponding table. (C) We also show compound characterizations for each of the 35 secondary metabolites. Where possible, we include tentative identifications of each compound, as well as retention time, diagnostic ions, exact mass, references, metabolomics confidence score, and mean fraction (±1 SD) base peak chromatogram ion counts per milligram of red alder leaf tissue. Note that this table is reproduced from the work of Jackrel et al. (10). Also note that all leaf chemistry analyses were completed on leaves collected during the 2012 growing season. We also used leaves from these same trees in reciprocal transplant leaf pack experiments that were designed to test whether decomposer communities decompose local leaves more rapidly than nonlocal leaves (i.e., home-field advantage). One experiment first reported in the work of Jackrel and Wootton (11) was completed in 2012 using leaves from the 2012 growing season. A second experiment was completed in 2013 using leaves from the 2013 growing season. Both experiments suggested a home-field advantage as calculated using the method reported by Ayres et al. (13) (2013: *t*_9_ = 2.60, *P* = 0.014; 2012: *t*_9_ = 1.96, *P* = 0.041). Download FIG S2, PDF file, 0.3 MB. Copyright © 2019 Jackrel et al. 2019 Jackrel et al. This content is distributed under the terms of the Creative Commons Attribution 4.0 International license.

10.1128/mBio.01703-19.9 FIG S8 As a supplement to Fig. 1 of the main text, here we show the corresponding decomposition measures as nonstandardized data points in which decomposition rates within an incubation site have not been adjusted to μ = 0, SD = 1. Categories on the *x* axis include, from right to left, trees growing immediately upstream of the incubation site and trees growing further upstream of that incubation site at the “Away Site” on the same river, both of which are considered “Home,” and then trees growing downstream of the incubation site at the “Away Site” on the same river, trees growing at the upstream site on the paired river, and trees growing at the downstream site on the paired river, all three of which are considered “Away.” Note that all points are horizontally jittered to minimize overplotting. Also note that leaf mass remaining can be above 100% because leaves absorb excess water when submerged in rivers. Download FIG S8, PDF file, 0.2 MB. Copyright © 2019 Jackrel et al. 2019 Jackrel et al. This content is distributed under the terms of the Creative Commons Attribution 4.0 International license.

**FIG 2:**
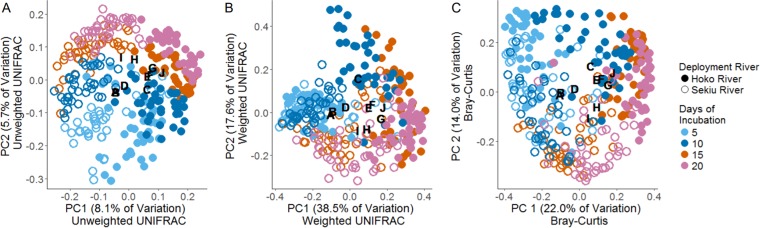
Successional changes among the aquatic bacterial community inhabiting and decomposing leaves from riparian red alder trees deployed in leaf packs on the streambeds of the Hoko and Sekiu Rivers, WA, USA. Community similarity is depicted using principal-coordinate analyses of either relative-abundance unweighted (A) or weighted (B) phylogenetic distance using the UniFrac metric, as well as the non-phylogenetically based Bray-Curtis distance metric (C). A biplot analysis depicts where the 10 most abundant taxa for all leaf pack samples, labeled taxa A to J, occur in principal-coordinate space to visualize successional changes among taxa. The earliest stage of decomposition, during day 5, is characterized by high relative abundance of taxa A and B, both in the order *Burkholderiales*, family *Comamonadaceae*. Mid-stage decomposition, during days 10 and 15, is characterized by increased relative abundance of taxa C (order *Myxococcales*), D (order *Sphingomonadales*, family *Sphingomonadaceae*, genus *Novosphingobium*), E (order *Rhodobacterales*, family *Rhodobacteraceae*, and genus *Rhodobacter*), and F (order *Sphingomonadales* and family *Sphingomonadaceae*). The last stage of decomposition, during day 20, was characterized by a high relative abundance of taxa G (order *Cytophagales*, family *Cytophagaceae*, genus *Flectobacillus*), H (order *Flavobacteriales*, family *Flavobacteriaceae*, genus *Flavobacterium*), I (order *Rhizobiales*, family *Rhizobiaceae*, and genus *Agrobacterium*), and J (order *Rhizobiales*).

We surveyed the bacterial community on leaves during decomposition as well as the bacterial community of the immediately surrounding environment to determine if the community composition corresponded with decomposition rate. The bacterial communities inhabiting the riparian soil, water column, terrestrial leaf, and aquatic leaf pack habitats each formed significantly distinct clusters from each other habitat in principal-coordinate space, when considering either the relative abundance-weighted or unweighted phylogenetically based community distance metric, UniFrac (Fig. S3; all pairwise analysis of similarity [ANOSIM] *P* < 0.01) (19). Using the Bayesian tool SourceTracker to predict the potential source of bacterial operational taxonomic units (OTUs) associated with each aquatic leaf pack, we found that aquatic bacterial communities on leaves were derived more from terrestrial leaves (66.7% ± 0.86% [standard error {SE}]) and unknown sources (29.6% ± 0.89%), while bacteria sourced from riparian soil and the river water column contributed marginally (0.82% ± 0.039% and 2.9% ± 0.14%, respectively) (20). These proportions varied considerably over time and by whether the leaves originated from Home or from Away sites; however, these results were site dependent as indicated by a significant day × leaf origin × site interaction in an analysis of variance (*F*_5,290_ = 4.3, *P* < 0.001 [Fig. 2A and B]). The proportion of bacteria from taxa that were sourced from terrestrial leaves tended to increase over the course of the experiment from an average of 59.0% ± 2.2% during day 5 to an average of 74.2% ± 0.7% during day 20 (ANOVA: *F*_1,290_ = 11.7, *P* < 0.001 [Fig. S4A and B]).

10.1128/mBio.01703-19.4 FIG S3 Community similarity illustrated using the phylogenetic distance metrics, unweighted (A) and weighted (B) UniFrac. Communities taxonomically described using 16S rRNA marker gene surveys cluster in principal-component space by habitat riparian soil, river water, and leaves taken either directly from riparian red alder trees or from red alder leaf packs submerged underwater on the streambed. All groups differ significantly from each other (all pairwise ANOSIMs, *P* < 0.01). (C) Further, we describe the relative abundance of bacterial taxa in summary tables for each environment. Despite containing thousands of rare taxa, environmental samples were dominated by relatively few particularly abundant taxa. Among aquatic leaves, note the decline in *Comamonadaceae* over time. In contrast to the community inhabiting these leaves, the adjacent water column samples remain largely stable over time. The first column shows the relative abundance in the water column, and the second column show the relative abundance on leaves. Taxa included in this table comprised at least 1% of the bacterial community averaged across all leaf packs samples for the noted time point. Download FIG S3, PDF file, 0.5 MB. Copyright © 2019 Jackrel et al. 2019 Jackrel et al. This content is distributed under the terms of the Creative Commons Attribution 4.0 International license.

10.1128/mBio.01703-19.5 FIG S4 Bacteria inhabiting red alder leaf packs submerged on riverbeds are derived more so from terrestrial leaves (estimated 66.7% ± 0.9% [SE]) (A) and unknown sources (29.6% ± 0.9%) (B), while the water column and riparian soil are not shown as they were more minor contributors (2.9% ± 0.1% and 0.82% ± 0.04%, respectively) to the bacterial community according to Bayesian SourceTracker models. Proportions of the bacterial community derived from terrestrial leaves varied significantly by days of incubation and leaf origin; however, effects were incubation site specific. Panels indicate site of incubation. Note that all points are horizontally jittered to minimize overplotting. Download FIG S4, PDF file, 0.1 MB. Copyright © 2019 Jackrel et al. 2019 Jackrel et al. This content is distributed under the terms of the Creative Commons Attribution 4.0 International license.

The greatest contributor to differences in bacterial community β-diversity was the river in which the leaf packs were incubated when considering a relative abundance-unweighted phylogenetic metric (Fig. 2A). In contrast, succession was the greatest contributor to β-diversity differences when considering either a relative abundance-weighted phylogenetic metric (Fig. 2B) or a nonphylogenetic metric (Fig. 2C). These contrasting results indicate that habitat location is the primary driver of OTU presence/absence, whereas successional stage is the primary driver of OTU relative abundance. Metrics incorporating relative abundance are usually more representative of community function; however, see the work of Jousset et al. (21) for a review of the potentially disproportionate role of rare taxa in ecosystem function. The Hoko River was characterized by a high relative abundance of taxa in the orders *Rhizobiales*, *Cytophagales* (family *Cytophagaceae*, genus *Flectobacillus*), *Sphingomonadales* (family *Sphingomonadaceae*), *Rhodobacter* (family *Rhodobacteraceae*, genus *Rhodobacter*), and *Myxococcales*. The Sekiu River was characterized by a high relative abundance of taxa in the orders *Burkholderiales* (family *Comamonadaceae*) and *Rhizobiales* (family *Rhizobiaceae*, genus *Agrobacterium*).

Early-stage decomposition was characterized by a very high relative abundance of taxa in the order *Burkholderiales* (family *Comamonadaceae*) (ANOVAs: taxon relative abundance predicted by day, all *P* < 0.001; see Fig. S5). OTUs in this family typically comprised over 50% of the early-stage community (Fig. S3). Mid-stage decomposition during days 10 and 15 was characterized by increased relative abundance of bacteria in the orders *Myxococcales*, *Sphingomonadales* (family *Sphingomonadaceae*, genus *Novosphingobium*), and *Rhodobacterales* (family *Rhodobacteraceae*, genus *Rhodobacter*) (*P* < 0.001 for all ANOVAs [Fig. S5]). Late-stage decomposition during day 20 was characterized by an increased relative abundance of taxa belonging to the orders *Cytophagales* (family *Cytophagaceae*, genus *Flectobacillus*), *Flavobacteriales* (family *Flavobacteriaceae*, genus *Flavobacterium*), and *Rhizobiales* (family *Rhizobiaceae*, genus *Agrobacterium*) (*P* < 0.001 for all ANOVAs [Fig. S5]). We provide a full list of taxa that change significantly in relative abundance over time in Fig. S5 where we categorize taxa as characteristic of early-, mid-, or late-successional communities based on visual inspection of relative abundance plots (*P* < 0.05 for all ANOVAs of taxon relative abundance predicted by day; see Fig. S5). We also separately list taxa with significant, but site-dependent, successional patterns that may be hypothesized to play a larger role in driving locally accelerated decomposition patterns (Fig. S5). Many of the taxa that became quite abundant in leaf packs during the later stages of decomposition remained exceedingly rare at less than 0.01% of the water column community (Fig. S3). We also found that leaves of Away origin were inhabited by communities richer in α-diversity than leaves of Home origin and that this difference in Faith’s phylogenetic diversity (PD) by leaf origin was consistent throughout stages of decomposition (Fig. S6, ANOVA: leaf origin, *F*_1,315_ = 2.39, ordered *a priori r_s_P_C_* < 0.01). Further, alpha-diversity varied by site (ANOVA: site, *F*_3,315_ = 1,942.5, *P* < 0.001) but remained stable over the course of succession (ANOVA: day, *F*_1,315_ = 0.30, not significant [NS]).

10.1128/mBio.01703-19.6 FIG S5 Certain taxa inhabiting decomposing alder leaf litter varied significantly in relative abundance over time. (A) We characterized each taxon as early, mid-, or late successional based on visual inspection of relative abundance plots, as shown below. We defined early-stage taxa as those with highest relative abundance during day 5, mid-stage as those with highest relative abundance during days 10 and 15, and late-stage taxa as those with highest relative abundance during day 20. We restricted this analysis to 91 taxa that comprised at least 1% of the community in at least one sample. Instead of averaging across our sample set, this approach included taxa that may comprise a sizable portion of a community, but only in a subset of samples. (B) We also summarize these results in a table where all reported significance values were corrected for multiple-comparison testing using the false-discovery rate correction. We also note taxa with significant day × site interactions, as well as taxa with significant day × leaf origin and day × leaf origin × site interactions to identify taxa that may be contributing to accelerated decomposition of local leaves. Only a single taxon in the order *Pedosphaerales* showed a significant day × leaf origin interaction. Download FIG S5, PDF file, 0.8 MB. Copyright © 2019 Jackrel et al. 2019 Jackrel et al. This content is distributed under the terms of the Creative Commons Attribution 4.0 International license.

10.1128/mBio.01703-19.7 FIG S6 Packs of leaves derived from riparian red alder trees growing a distance away from the incubation site tended to harbor greater bacterial diversity than leaf packs consisting of leaves from the immediately local riparian zone. A linear mixed model of Faith’s phylogenetic diversity against leaf origin was conducted on five categories of leaf origin with an ordered-ANOVA correction *r_s_P_C_* to test our *a priori* hypothesis that leaves in the Home categories would differ from leaves in the Away categories. For illustration, we show this condensed contrast of Home versus Away that includes all five leaf origin categories reduced to two. Alpha-diversity metrics are shown here as standardized scores (i.e., z-scores), in which diversity measures within an incubation site and by each day are adjusted to μ = 0, SD = 1, so that a *y* axis value of 1 indicates 1 SD above the mean alpha-diversity measurement for that incubation site from that day. This standardization serves to illustrate the relative diversity measures of the Home versus Away leaf communities; however, the mixed-effects model was run on the nonstandardized data with day as a fixed effect and incubation site and tree identity as a random effect. Note points are horizontally jittered to minimize overplotting. Download FIG S6, PDF file, 0.3 MB. Copyright © 2019 Jackrel et al. 2019 Jackrel et al. This content is distributed under the terms of the Creative Commons Attribution 4.0 International license.

**FIG 3:**
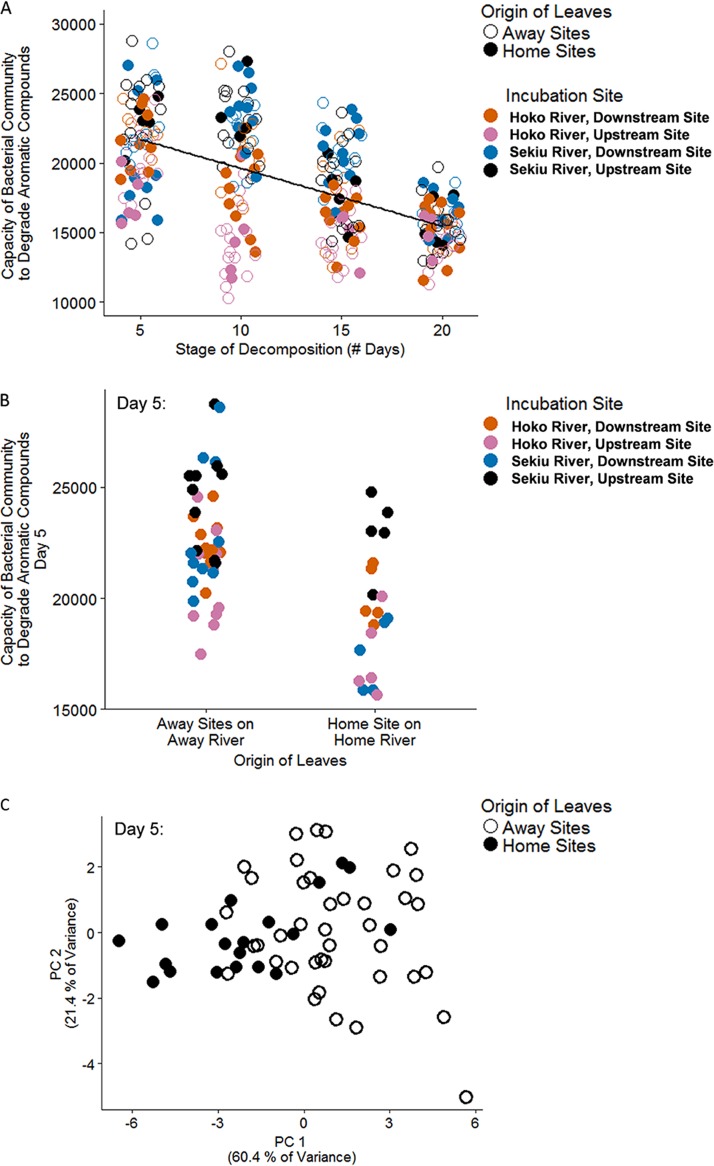
(A) Decline in predicted aromatic degradation ability of the bacterial community during leaf succession (*F*_1,157_ = 313, *P* < 0.001, *R*^2^ = 0.33). (B) Differences of ability as a function of leaf source and incubation locations early in succession (ANOVA: site *F*_3,52_ = 11.8, *P* < 0.001; leaf origin *F*_1,52_ = 32.6, *P* < 0.001). Note that for panels A and B, *y* axis units are gene content predictions as calculated via PICRUSt, and points are horizontally jittered to minimize overplotting. (C) In addition to differences in the summed capacity to degrade aromatics, early-successional bacterial communities (i.e., day 5) differed as a function of leaf origin in the relative abundance of different enzymes that contribute to the summed capacity to degrade aromatic ring structures. We defined “summed capacity” as that of all KEGG terms included in the pathway that were represented in our data set to obtain a total measure of pathway function per sample. Figure S7 gives principal-coordinate (PC) axis loadings. Note that panel A included all leaf origin categories, whereas panels B and C consider only the strongest contrast: Home Site on the Home River versus Away River sites.

We next analyzed changes in the predicted metabolic functional capacity of bacterial communities both over succession (i.e., day) and by leaf origin. Our predicted metagenome functions are derived from bacterial taxa with reasonably short evolutionary distances to yield accurate predictions (average nearest sequences taxon index < 0.10, validated by Langille et al. [22]). Bacterial communities that inhabited leaves during the earliest stage of decomposition had the greatest predicted capacity to degrade aromatic compounds, with a significant decline in predicted capacity through succession (Fig. 3A) (*R*^2^ = 0.33, *P* < 0.001, gene content predictions for day 5 = 21,566 ± 365 [mean ± SE], day 10 = 19,826 ± 497, day 15 = 17,702 ± 365, day 20 = 15,284 ± 187). During the earliest stage of decomposition, day 5, when the relative abundance of these taxa was greatest, we found that communities inhabiting leaves of Away origin contained a greater proportion of these taxa than communities inhabiting leaves of Home origin (Fig. 3B) (ANOVA: leaf origin, *F*_1,52_ = 32.6, *P* < 0.001). Further, Home versus Away leaf origin predicted the relative abundance of 12 molecular functional terms within the aromatic degradation pathway (ANOVA: leaf origin, all *P* values < 0.05 after false-discovery rate correction for multiple comparisons). For an illustration of the role that each of these 12 molecular functional terms play within this pathway, see Fig. S7. We found that the bacterial communities inhabiting leaves of Home versus Away origin differed significantly in the predicted relative abundance of a mixture of enzymes required for degrading aromatic compounds (Fig. 3C). The more abundant taxa predicted to be capable of degrading aromatic compounds that contributed most toward these functional measures belonged to two families, the *Comamonadaceae*, which contributed 45.3% summed across all leaf samples of gene content corresponding to aromatic degradation and *Sphingomonadaceae*, which contributed 31.0%. Other families that contributed substantially to these predicted functions were *Oxalobacteraceae* (3.5%), *Rhizobiaceae* (3.4%), *Rhodobacteraceae* (3.2%), *Caulobacteraceae* (1.7%), *Phyllobacteriaceae* (1.2%), *Hyphomicrobiaceae* (1.1%), *Neisseriaceae* (1.1%), and *Cytophagaceae* (1.1%). An additional 49 families contributed less than 1.0% toward the total predicted gene content for aromatic degradation.

10.1128/mBio.01703-19.8 FIG S7 (A) Illustration of the subset of pathways in the “degradation of aromatic compounds” pathway that involve the 12 molecular functional terms that were identified as key differences between the bacterial communities inhabiting Home versus Away leaves using principal-component analyses. See the principal-component analysis in Fig. 3C and panel B for further description of the 12 molecular functional terms. The corresponding table lists all the KEGG Ontology molecular functional terms included in the two pathways included in our analyses using PICRUSt metagenome functional predictions. The 12 boldface terms in the “degradation of aromatic compounds” pathway were key in distinguishing between bacterial communities inhabiting leaves of different leaf origin, while the 8 boldface terms in the “metabolism of starch and sucrose” pathway are those that we studied further for their involvement in cellulose degradation. (C) Further, we report factor loadings for each of these 12 boldface terms in the “degradation of aromatic compounds” pathway that were used as variables in a principal-component analysis that illustrates the distinct bacterial communities inhabiting Home versus Away leaves. Enzyme commission (EC) numbers provide a nomenclature reference, and the Description column gives commonly used names. Download FIG S7, PDF file, 0.6 MB. Copyright © 2019 Jackrel et al. 2019 Jackrel et al. This content is distributed under the terms of the Creative Commons Attribution 4.0 International license.

**FIG 4:**
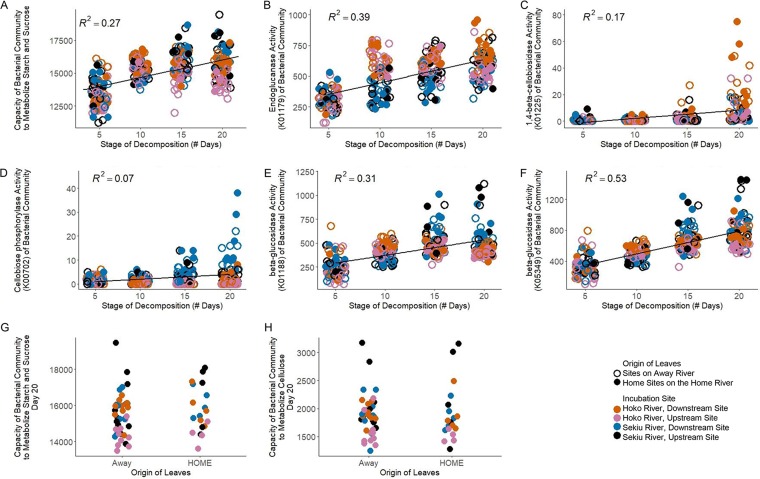
Successional patterns of starch and sucrose metabolism pathways in the bacterial community. (A) Overall predicted functional capacities from all molecular functional terms, including enzymes known to degrade the plant polysaccharide cellulose, such as endoglucanase and cellulose 1,4-beta-cellobiosidase increase over time *F*_1,313_ = 120.6, *P* < 0.001. (B to F) Prevalence of 5 enzymes involved in cellulose breakdown that increase significantly with succession. (G and H) Total predicted functional capacity to degrade starch and sucrose (G) and total predicted functional capacity to degrade cellulose (H) do not vary as a function of leaf origin during the latest stage of leaf decomposition, day 20. Note that panels A to F included all leaf origin categories, whereas panels G and H considered only the strongest contrast: Home Site on the Home River versus Away River sites. Also note that all *y* axis units are gene content predictions as calculated via PICRUSt, and all points are horizontally jittered to minimize overplotting.

In addition to focusing specifically on leaf secondary metabolites, we also evaluated the effect of succession (i.e., day) and leaf origin on the more general degradation of leaf material. As decomposition progressed, the bacterial community became increasingly dominated by taxa with the predicted functional capacities categorized in the “metabolism of starch and sucrose” pathway (*R*^2^ = 0.27, *P* < 0.001 [Fig. 4A; see Fig. S7 for specific molecular functional terms included in this pathway]). This diverse pathway includes the enzymes known to degrade the plant polysaccharide cellulose, including endoglucanase (K01179) and cellulose 1,4-beta-cellobiosidase (CBH1) (K01225). In addition to this broader category of “metabolism of starch and sucrose,” we highlight five specific molecular functional terms implicated in cellulose degradation that increased significantly over succession (Fig. 4B to F). However, during the final stages of decomposition, day 20, we did not find differences in the starch and sucrose predicted metabolic capacities of bacteria inhabiting leaves of Home versus Away origin, either when summing function across the entire pathway (Fig. 4G) or in our cellulose-specific pathway of these five molecular functional terms (Fig. 4H).

Last, we aimed to determine an adequate minimal model for predicting the rate of leaf decomposition by considering the relative importance of leaf bacterial diversity, bacterial predicted metabolic function, and our leaf origin treatment. Considering evidence that biological diversity often promotes ecosystem functions (23), we hypothesized Faith’s phylogenetic diversity (24) might promote more rapid leaf decomposition. Further, we included the second two terms in the model because plant defense compounds are thought to be toxic and/or repellent toward decomposers as a means of preventing decomposition of nondefended components of the plant tissue. Therefore, we hypothesized that rapid decomposition may occur among local leaves with specialized bacterial communities predicted to have a relatively high capacity to degrade aromatics in early stages of decomposition, followed by communities predicted to have a high capacity to degrade cellulose during later stages of decomposition. We found that the phylogenetic diversity and predicted functional composition of the bacterial community were significantly predictive of the rate of leaf decomposition (Faith’s PD *F*_1,8_ = 3.3, predicted cellulose degradation *F*_1,8_ = 1.0, predicted aromatic degradation *F*_1,8_ = 6.7, marginal *R*^2^ = 0.057). This model incorporating bacterial diversity and predicted functional patterns explains nearly as much variance as a model including our leaf origin treatment as the sole predictor of decomposition rate (leaf origin *F*_4,9_ = 14.7, marginal *R*^2^ = 0.064). The leaf origin model fit the data significantly better than the bacterial model, as determined with a log-likelihood test of Akaike information criterion (AIC) scores. However, considering all measures together generated the best-fitting model; adding our metrics of bacterial alpha-diversity and metabolic function significantly improved the leaf origin model (leaf origin *F*_4,12_ = 15.0, Faith’s PD *F*_1,8_ = 4.3, predicted cellulose degradation *F*_1,8_ = 1.3, predicted aromatic degradation *F*_1,8_ = 3.2, marginal *R*^2^ = 0.15). We also used a second approach to determine the relative importance of bacterial diversity, predicted functional metabolism, and leaf origin on decomposition rate. We summarized our predictor variables as principal components and used these principal components in a model as composite variables predictive of decomposition rate. These results generated similar conclusions that leaf origin is a stronger predictor of decomposition rate than bacterial measures, but bacterial community composition further improves prediction in the final model (PC2 with loadings on bacterial predicted functional metabolism *F*_1,311_ = 13.5; PC3 with loadings on leaf origin *F*_1,311_ = 28.5 [Table S1]).

10.1128/mBio.01703-19.10 TABLE S1 (A and B) To determine the relative importance of bacterial diversity, predicted functional metabolism, and leaf origin on decomposition rate, we summarized our predictor variables as principal components (A) and used these principal components in models as composite variables predictive of decomposition rate (B). (C) We show the analysis of variance table from the best-fitting model. Download Table S1, PDF file, 0.1 MB. Copyright © 2019 Jackrel et al. 2019 Jackrel et al. This content is distributed under the terms of the Creative Commons Attribution 4.0 International license.

## DISCUSSION

Our results suggest that we can begin to characterize the role of particular freshwater bacterial taxa in degrading allochthonous leaf litter in streams. We found similar patterns of successional change across leaf packs from multiple trees in each of our two study rivers. A core generality emerged from the patterns of succession that we documented: microbial taxa specialized in breaking down aromatic secondary metabolites of leaves dominated early stages of succession, whereas taxa that efficiently break down more generalized plant tissue components, such as cellulose and starches, dominate later stages. Studies of microbial decomposition of both plants and animals increasingly find clearly defined stages of microbial succession (25–28). For example, bacterial succession on mammalian corpses is sufficiently consistent across environments to warrant use of the microbial community for forensic predictions (26). We found that the most relatively abundant taxa during each stage of decomposition tended to be rare in the water column, which agrees with previous findings that key taxa involved in animal decomposition are initially exceedingly rare but widespread in terrestrial soils across environments (26). Succession was the primary source of variation among freshwater bacterial communities when weighting by relative abundance of each taxon. However, the river during incubation was the primary source of variation when considering non-relative-abundance-weighted communities. Regardless of using a weighted or nonweighted metric of relative abundance, leaf origin was responsible for a smaller, yet statistically significant, proportion of the variation among bacterial communities. Prior studies of the freshwater microbial communities inhabiting leaves from different genotypes of cottonwood trees also found that the environment was a more substantial driver of community variation than leaf traits (29).

Beyond expanding our understanding of bacterial successional patterns that drive decomposition of leaves in rivers, we find evidence of the mechanisms by which freshwater bacteria may be accelerating the decomposition of local leaves. Specifically, this evidence supports our first hypothesis that leaves of local origin will harbor a more specialized community of aquatic bacteria over the course of decomposition that is better able to rapidly break down the abundant local food source. Leaf secondary metabolites, which are often toxins targeting herbivores and microbial enemies (30, 31), vary among the Alnus rubra trees studied here. Although not necessarily targeting decomposing microbes, these toxins must nonetheless be neutralized before the nontoxic leaf components are accessible to the broader microbial and invertebrate decomposer community. We found that during early succession, leaves derived from local versus nonlocal trees were inhabited by significantly different communities. It is intuitive that we would find our most striking differences between Home and Away leaves at the earliest stages of decomposition because while leaf secondary metabolites are diverse and structurally complex, they are constructed of many of the same building blocks that ultimately degrade to a small number of simple sugars that are accessible to many microbial taxa (however, it should be noted that studies have also shown that the magnitude of differences between Home and Away leaves may increase over the course of decomposition in some terrestrial environments [32]). During the earliest stage of decomposition, freshwater bacterial inhabitants are likely to be specialists capable of degrading intact secondary metabolites that we have shown to vary spatially in relative abundance across this landscape (see Fig. S2 in the supplemental material) as well as other aromatic compounds such as lignin. Predicted functional annotation revealed that leaves were inhabited early by taxa capable of degrading aromatic compounds and that this metabolic capacity to degrade aromatic compounds differed between Home and Away leaves. We therefore infer that these taxa are involved in locally accelerating decomposition. We base this inference on our prior work showing that Home and Away leaves differ in their composition of aromatic compounds and that these differences drive leaf decomposition rates (10). Further supporting our first hypothesis that leaves of local origin will harbor a more specialized community of aquatic bacteria, our results suggest that decomposition in streams of nonlocal leaves requires a more diverse freshwater bacterial community than local leaves. We found that across different stages of succession, Away leaves tended to be inhabited by more diverse communities than Home leaves. Our metabolic functional predictions further suggest that these Away communities include many specialist taxa with the capacity to degrade aromatic compounds.

We focused our study on bacterial degradation of aromatic compounds because we had previously documented intraspecific variation in *A. rubra* leaves in several classes of aromatic secondary metabolites (i.e., ellagitannins and diarylheptanoids [see Fig. S2 and reference 10]). However, in addition to these aromatic defensive compounds, lignin is a ubiquitous aromatic polymer that lends structure to plant cell walls. Degradation of plant litter at both early and late stages of decomposition is regulated in part by lignin (33), and while studies of lignin decomposition have focused mostly on degradation pathways in fungi, more recent studies have reported that lignin degradation pathways also exist in some bacterial taxa (34). Further studies are necessary to disentangle the relative importance of lignin versus other classes of aromatic secondary metabolites in regulating bacterial community composition, bacterial metabolism, and rates of litter decomposition. However, knowing the precise makeup of aromatic compounds in each individual tree at the time of the experiment is not necessary to conclude that divergence in taxa capable of degrading aromatic compounds between Home and Away leaves is involved in locally accelerating decomposition.

We also found evidence in support of our second hypothesis, that the aquatic bacterial community inhabiting local leaves will undergo more rapid successional change than communities inhabiting leaves of nonlocal origin. Specifically, we found that Away communities appear to have a greater capacity for aromatic degradation at day 5, possibly indicating that Away leaves require a greater relative abundance and/or longer residence time of taxa capable of metabolizing locally novel plant secondary metabolites. Future studies that are more temporally resolved during this day 0 to 5 period would help clarify whether Away leaves have a greater abundance and longer residence time of taxa capable of degrading aromatics or whether Home leaves might have had equivalent or even greater capacity for aromatic degradation than Away leaves at some time prior to day 5. As these bacteria degrade plant secondary metabolites, later stages of succession may then sustain other bacterial taxa that can thrive on the subunits of the original plant metabolites, such as the successional patterns observed from primary to secondary consumers on marine particles (25). During later stages of decomposition, we found that freshwater bacterial communities no longer consisted largely of specialized taxa capable of degrading plant secondary metabolites but instead were dominated by generalists with enzymes capable of degrading cellulose, cellobiose, and simple carbohydrates.

The freshwater bacterial communities inhabiting leaves during the early stages of decomposition were dominated by two taxa of bacteria that are especially known for their capacity to degrade aromatics, the *Burkholderiales* and *Sphingomonadaceae* (35). Taxa within multiple families of *Burkholderiales* harbor genes that are key for the degradation of aromatics (36). Bacteria in the genus *Burkholderia* are especially important degraders of aromatic pollutants, and single strains can have multiple pathways for their degradation (37–39). The most abundant *Burkholderiales* taxa in our communities belonged to the *Comamonadaceae*, particularly *Comamonas* spp. Genome surveys of taxa within *Comamonadaceae* found that half of the taxa harbor dioxygenase genes required for the degradation of protocatechuate (36). Our results suggest that metagenome-level investigations of the *Burkholderiales* taxa in our study system may be warranted to help elucidate the role of these metabolic functions in locally accelerated decomposition. One could investigate whether the genetic architecture of these bacterial taxa explains their capacity of accelerated decomposition of local leaves, such as higher inherent rates of mutation or more rapid generation time. Further, plant polyphenols, such as the ellagitannins and diarylheptanoids common in red alder, are structurally analogous to anthropogenic, persistent polycyclic aromatics (6). Bioremediation strategies often take advantage of this structural similarity by using plant secondary metabolites to boost the activity of microbes that can cometabolize both anthropogenic and natural aromatic hydrocarbons (6). Bacterial taxa that are fine tuned to spatial variation in plant secondary metabolites could perhaps be applied toward experimental evolution of improved degradation of anthropogenic aromatic pollutants (40).

We found that the bacterial communities of the terrestrial leaf phyllosphere partly contributed to the community that inhabited aquatic leaf packs using SourceTracker predictive models. We found that these taxa comprised an increasingly large portion of the leaf community later in succession, suggesting that populations of these taxa originating from the terrestrial environment tended to grow more than populations of taxa originating from the other source environments. Further study into how the phyllosphere would drive locally accelerated decomposition may be warranted. The living phyllosphere should travel within the leaf to the location of decomposition, where facilitative and/or antagonistic interactions with resident aquatic bacteria may then affect rates of decomposition. While it is challenging to explain how the home-field advantage might arise when most of the bacterial decomposer community originates from the phyllosphere, several fungus-based studies reach similar conclusions that fungal decomposers in soils are largely derived from the fungal community residing within the living phyllosphere (12, 41, 42). Unknown sources also contributed to the bacterial community inhabiting aquatic leaf packs. Unknown sources could include an environment that was not sampled, such as stream sediments. Alternatively, rare taxa residing in the sampled environments but below the level of detection might contribute to this unknown fraction. This might be especially common in our experiment because we surveyed varied habitats that would select for different bacterial physiologies. For example, a rare bacterium inhabiting the water column at a relative abundance below the level of detection may grow rapidly in population size once settling on a submerged leaf. Including quite different habitat types as potential sources may therefore limit predictions.

There are several limitations to our study. First, bacterial 16S rRNA surveys yield community composition data in terms of relative, not absolute, abundance. The limitations of relative abundance data are well appreciated, but acquiring absolute abundance measures remains challenging, particularly for bacterial communities inhabiting more complex environments. Quantities of extracted DNA are not suitable proxies for bacterial abundance due to many factors that influence extraction efficiency, such as leaf inhibitory compounds within leaf tissue. Newer methods for quantifying absolute abundance are actively being developed to overcome this limitation (43, 44). Second, an in-depth survey of temporal changes within bacterial communities came at the expense of broader spatial scope. We chose four sites within two study rivers close to each other because this proximity is what makes the observed patterns of locally accelerated decomposition particularly notable. While we have found similar patterns of locally accelerated decomposition in a second pair of rivers, without matching data in other systems, we caution that further studies are needed before generalizing these results more broadly to other river systems. Third, while our aim was to probe for the role of bacteria in degrading leaf secondary metabolites, other aromatic compounds are undoubtedly produced by decomposers. To infer a direct link of specific microbes to degrading plant secondary compounds, additional types of data are needed such as experiments using isotopically labeled plant secondary metabolites and metagenome surveys of bacteria and fungi. Further, it is important to note that our study used fresh green leaves rather than senescent leaf litter. These two types of leaf litter play key but different roles in stream nutrient cycles and food webs. Although some forested streams may receive minimal greenfall (less than 2.5% of total leaf fall [45]), some temperate rainforest streams can receive as much as ∼20% of their annual leaf flux as greenfall (46). Although a smaller proportion of annual leaf fall, green leaf litter has greater nutrient content, including nitrogen, phosphorus, and potassium (45, 47), as well as a lower lignin-to-nitrogen ratio (48). This more nutrient-dense food source, which enters streams during the summer growing season for many aquatic organisms, can have critical implications for stream productivity. For example, despite the greater abundance of autumnal leaf litter, phosphorus inputs from leaf litter per square meter of stream have been reported as 4 times higher in summer due to this higher nutrient content of green leaves (47). Further, when these fresh, nutrient-dense leaves are available, aquatic macroinvertebrates grow more rapidly than when only senescent leaves are available (49, 50). Further investigation into how nutrient cycles differ between streams receiving different proportions of leaf fluxes as greenfall is warranted. Although we do not have data on the secondary chemistry or decomposition patterns of senescent red alder leaves, given known differences between green and senescent litter reported elsewhere (51), testing whether our findings using green leaves apply when using senescent leaf litter would be a valuable future direction.

We also note that these results should also be considered in light of limitations inherent in using the PICRUSt tool. Metagenome predictions rely on the accuracy of 16S rRNA sequencing data to depict a microbial community. Primer biases can lead to inaccurate descriptions of bacterial communities, as well as omit the viral and eukaryotic components of microbial communities. Further, PICRUSt predictions are limited by the depth and accuracy of available databases. Genes lacking sufficient annotation may play critical functional roles but would go undetected with this approach. Further, a limitation of PICRUSt, particularly when using environmental samples, is that the tool predicts gene content using reference genomes. Therefore, accurate predictions depend on the availability of appropriate references (note: we have shown that our environmental samples have appropriate references by finding an average nearest sequences taxon index of <0.10). Despite these limitations, our results begin to shed light on the complex bacterial component of leaf decomposition in rivers.

Ninety percent of terrestrial plant biomass enters the detrital food webs of soils and freshwater ecosystems (52). Understanding biotic controls on rates of leaf decomposition is important as these rates affect the proportion of organic carbon that is locally metabolized and respired as CO_2_, sequestered in the streambed, or exported further downstream. When paired with prior knowledge in this study system, the present study highlights how synchrony between leaf chemistry and local bacterial decomposers arises at small spatial scales and that disturbance of this synchronization significantly affects microbial community composition and function and, as a consequence, rates of decomposition. How these findings translate to predictions of long-term disturbance effects on carbon dynamics in riparian systems requires a better understanding of the underlying processes governing microbial community assembly, selection, and dispersal.

A key challenge is identifying mechanisms that may promote resilience of ecosystem function in the face of environmental change. Microbial components may offer important contributions to ecosystem resilience through their wide-ranging biochemical pathways and their high physiological, generational, and evolutionary rates. Our findings documenting changes through time and fine-scale environmental matching lend strong support to the notion that microbial community components can play a central role in stabilizing ecosystem function in a changing environment.

## MATERIALS AND METHODS

### Study site.

We studied decomposition of leaf litter in two rivers of the Olympic Peninsula of Washington State, USA. The Sekiu and Hoko Rivers are fourth-order streams with riparian forest comprised mostly of red alder (Alnus rubra), small numbers of bigleaf maple (Acer macrophyllum), and few conifers. We identified two reaches per stream that we refer to as upstream and downstream because their orientation relative to one another is important for the study design; however, all of the study reaches are relatively far downstream near the river mouths. Additional details regarding location, river morphology, and environmental characteristics can be found in reference 11.

### Field experiment.

We previously found that red alder trees vary geographically in leaf traits that strongly influence the rate of leaf decomposition in riparian soils and rivers (10). Here, we repeated our test of the effects of individual variation on leaf litter decomposition in streams, while simultaneously testing the effects of this variation on the composition of the bacterial decomposer community and the metabolic pathways associated with that community. We conducted a reciprocal transplant experiment of red alder leaf packs across two rivers in August 2013. The Hoko and Sekiu Rivers are approximately 4.5 km apart. Our upstream and downstream sites within each river were less than 1 km apart. We identified 10 trees in the riparian zones of the Sekiu River and 10 trees in the riparian zone of the Hoko River. Within each river, five riparian red alder trees were growing immediately upstream of the “upstream” incubation site, and five trees were growing immediately upstream of the “downstream” incubation site. Our incubation sites were at the most downstream section of each study reach, so that leaves from all riparian trees identified at that study reach would float downriver toward the incubation site (see Fig. S1 in the supplemental material for an illustration of the study design). Within each study reach, we deployed leaf packs onto the streambed using steel reinforcement bars.

For our experiment, we used only fresh green leaves to construct our leaf packs. Compared to brown, senescent leaves, green leaves decompose rapidly in streams, support high aquatic invertebrate diversity, and fall into rivers in large quantities during the summer growing season (49). We found that over 60% of red alder leaf litter is still green upon entering rivers of the Olympic Peninsula of Washington in summer (11). In the Hoko River, this equated to 96 g · m^−1^ · day^−1^ of green leaf material from red alder trees entering the river in July and August of 2012. We chose to use green leaves because our finding that intraspecific variation in *A. rubra* secondary chemistry causes local leaves to decompose more rapidly was done using green leaves. Whether a home-field advantage pattern occurs using senescent leaves of *A. rubra* has not yet been tested. We manually detached green leaves for use in our experiments rather than collect greenfall to enable collection of a large number of leaves that had been detached from their parent tree for a standardized amount of time, as well as to control for tree of origin.

From each red alder tree, we constructed four replicates of leaf packs that we deployed at each of the four incubation sites, including the “Home Site” that was immediately downstream of the source tree, the “Away Site” on the same river where the source tree was growing, and the “Away River, Upstream” and “Away River, Downstream” sites on the paired study river. Each of these leaf packs contained 16 leaves from a single alder tree. Twelve of these leaves were preweighed and dedicated to determining percent leaf mass loss. A mesh bag of these 12 leaves was placed into a second outer mesh bag that contained the remaining four leaves used for bacterial sequencing. We used only leaves with minimal visible damage from herbivores and disease. We collected one leaf from each of the outer mesh bags of each leaf pack after 5, 10, 15, and 20 days of incubation and sealed each leaf individually in a sterile Whirl-Pak bag. The remaining 12 leaves from each inner mesh bag of each leaf pack were blotted dry with paper towels and weighed to measure percent leaf mass remaining after 21 days of incubation. Although these inner leaf packs lost at most only a third of initial leaf mass over the 21-day incubation (maximum lost = 29.6%, mean lost = 10.6% ± 0.8% [SE]), it is important to note that this is not representative of the amount of leaf mass lost by leaves collected on day 20 for bacterial surveys. Leaves collected for bacterial surveys were from the outer mesh bags of the leaf pack, which decomposed considerably more than inner leaves. By 20 days, leaves in the outer leaf pack that were used for 16S rRNA sequencing were in the later stages of decomposition as indicated by a thin, skeletonized leaf completely black in color with little remaining structure. Based on our prior leaf pack studies in this system, we estimate our day 20 leaves would have disintegrated entirely within 1 to 5 extra days of incubation.

We sampled the freshwater bacterial community at each incubation site immediately prior to deploying our leaf pack experiment, as well as prior to leaf collections on days 5, 10, 15, and 20. Each sample consisted of up to 6 liters of river water pumped through a Sterivex filter (EMD Millipore, Darmstadt, Germany) with a peristaltic pump. Immediately before the 20-day experiment, we collected riparian soil samples beneath each source tree and green fresh leaves from each tree. All samples were frozen at −20°C immediately upon returning from the field locations and then stored at −80°C until processing.

### Bacterial sequencing.

We extracted DNA from all soil, water, and leaf samples using PowerSoil DNA isolation kits (Mo Bio Laboratories, Carlsbad, CA, USA). We used the identical extraction protocol for all samples to facilitate comparisons within our data set, as well as to meet the goals of the broader Earth Microbiome Project research collaborative to use standardized methods to best facilitate data syntheses. For water samples, Sterivex casings were cut with polyvinyl chloride (PVC) cutters and half of the filter paper was removed and then ground and extracted as a solid sample. We amplified the 254-bp length V4 region of extracted DNA using the Earth Microbiome Project universal primers (515F primer and 806 GoLay-barcoded reverse primers) (53). We chose not to use mitochondrial and chloroplast blocking peptide nucleic acid (PNA) clamps during PCR amplification due to our prior finding that use of the chloroplast pPNA sequence biases amplification of certain bacterial taxa (54, 55). We sequenced DNA fragments in a HiSeq 2500 2- by 151-bp run at the Environmental Sample Preparation and Sequencing facility at Argonne National Laboratory according to the procedures of Caporaso et al. (53). In brief, all bacterial sequencing data were analyzed using the QIIME pipeline. We assigned taxonomy to all 16S rRNA sequences using the Greengenes database, which was preferable for our study because of its compatibility with the functional annotation tool PICRUSt (Phylogenetic Investigation of Communities by Reconstruction of Unobserved States) (22). We used this PICRUSt software package to predict metagenome functional content by using ancestral-state reconstruction as a means of predicting the presence of gene families. We then used the Kyoto Encyclopedia of Genes and Genomes (KEGG) database to identify genes within certain functional pathways that we had hypothesized might play key roles in leaf degradation (i.e., pathways for the degradation of aromatic compounds, starch, sucrose, and cellulose). To predict where bacterial communities that inhabited our leaf packs originated, we used Bayesian SourceTracker models with a uniform prior for each of our known source environments (i.e., riparian soil, terrestrial leaves, and the water column) (20). For details of all data analyses, see Text S1 in the supplemental material.

10.1128/mBio.01703-19.1 TEXT S1 Supplemental methods. Download Text S1, PDF file, 0.2 MB. Copyright © 2019 Jackrel et al. 2019 Jackrel et al. This content is distributed under the terms of the Creative Commons Attribution 4.0 International license.

### Data accessibility.

All sequencing data have been permanently deposited at https://www.ncbi.nlm.nih.gov/Traces/study/?acc=PRJNA525284 (accession no. PRJNA525284).
