# Supplementary material for: The Origin, Succession, and Predicted Metabolism of Bacterial Communities Associated with Leaf Decomposition
Source: mBio. 2019 Sep 3;10(5):e01703-19. doi: 10.1128/mBio.01703-19 (PMC6722416; doi:10.1128/mBio.01703-19)
Supplement: FIG S1 [file mBio.01703-19-sf001.pdf]

ELECTRONIC SUPPLEMENTARY MATERIALS

Fig. S1. Illustration of experimental design for the reciprocal transplant experiment. Also listed are measurements of temperature, photosynthetically active radiation (PAR), flow rate, conductivity, pH and dissolved oxygen from July 2013 for each of the four sites used in the reciprocal transplant experiment. Measures of temperature and PAR are means over 7 day periods, except for the Sekiu upstream site (2 days). Daytime temperatures were determined from 10 min interval readings using HOBO data loggers for all non-zero light measurements. Maximum and minimum temperatures include daytime and nighttime readings. Flow rates were measured during 0.5 min increments using a Global Water flow probe. Dissolved oxygen, conductivity, and pH were measured using a Hach HQ40d multiprobe.

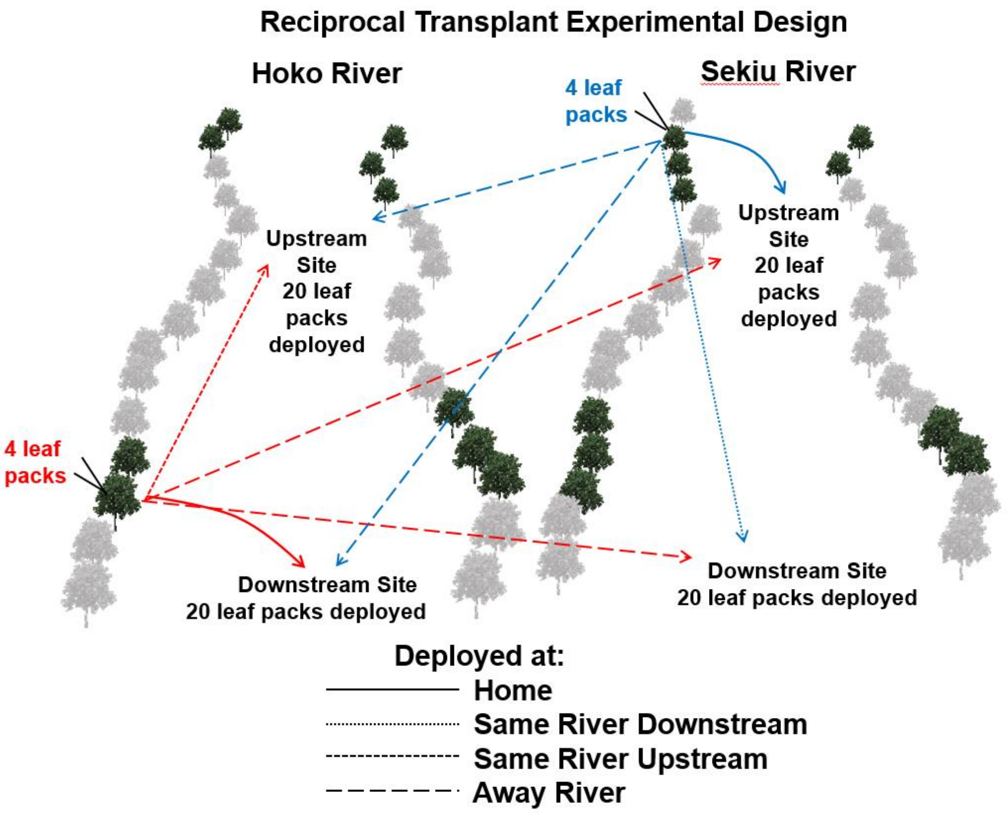

River:	Sekiu		Hoko	
Upstream; Downstream	Up	Down	Up	Down
Latitude (48°N+ °)	0.165	0.165	0.153	0.154
Longitude (124°W+ °)	0.245	0.244	0.211	0.211
Average temperature (°C)	16.1	15.9	16.7	16.2
Minimum temperature (°C)	14.1	13.9	14.1	13.9
Maximum temperature (°C)	18.2	18.9	19.8	18.8
PAR (µmol $m^{-2}s^{-1}$)	5677	8478	8304	18567
Width (m)	24.6	16.5	12.8	23.3
Depth (cm)	46	35	36	30
Flow rate (cm/s)	1.75	1.43	4.14	1.86
pH	5.82	5.75	5.88	5.9
Conductivity (µS/cm)	74.2	72.7	80.5	75.3
Dissolved oxygen (mg/L)	8.48	8.91	9.61	9.39
