# Supplementary material for: The Origin, Succession, and Predicted Metabolism of Bacterial Communities Associated with Leaf Decomposition
Source: mBio. 2019 Sep 3;10(5):e01703-19. doi: 10.1128/mBio.01703-19 (PMC6722416; doi:10.1128/mBio.01703-19)
Supplement: FIG S2 [file mBio.01703-19-sf002.pdf]

## Electronic Supplementary Materials

**Fig. S2.** (A) Leaves of individual red alder trees growing in the riparian zones of two rivers vary in the relative abundance of 35 secondary metabolites, including ellagitannins and diaryheptanoids, by tree origin via discriminant function analysis. (B) Loadings of each chemical variable, with tentative chemical ID, are shown in the corresponding table. (C) We also show compound characterizations for each of the 35 secondary metabolites. Where possible, we include tentative identifications of each compound, as well as retention time, diagnostic ions, exact mass, references, metabolomics confidence score, and mean fraction (± 1 SD) base peak chromatogram ion counts per milligram of red alder leaf tissue. Note that this table is reproduced from Jackrel et al. 2016. Also note that all leaf chemistry analyses were completed on leaves collected during the 2012 growing season. We also used leaves from these same trees in reciprocal transplant leaf pack experiments that were designed to test whether decomposer communities decompose local leaves more rapidly than non-local leaves (i.e. Home-Field Advantage). One experiment first reported in Jackrel and Wootton (2014) was completed in 2012 using leaves from the 2012 growing season. A second experiment was completed in 2013 using leaves from the 2013 growing season. Both experiments suggested a home-field advantage as calculated using the method reported by Ayres et al. 2009 (2013: $t_9 = 2.60$, $p = 0.014$, 2012: $t_9 = 1.96$, $p = 0.041$).

(A)

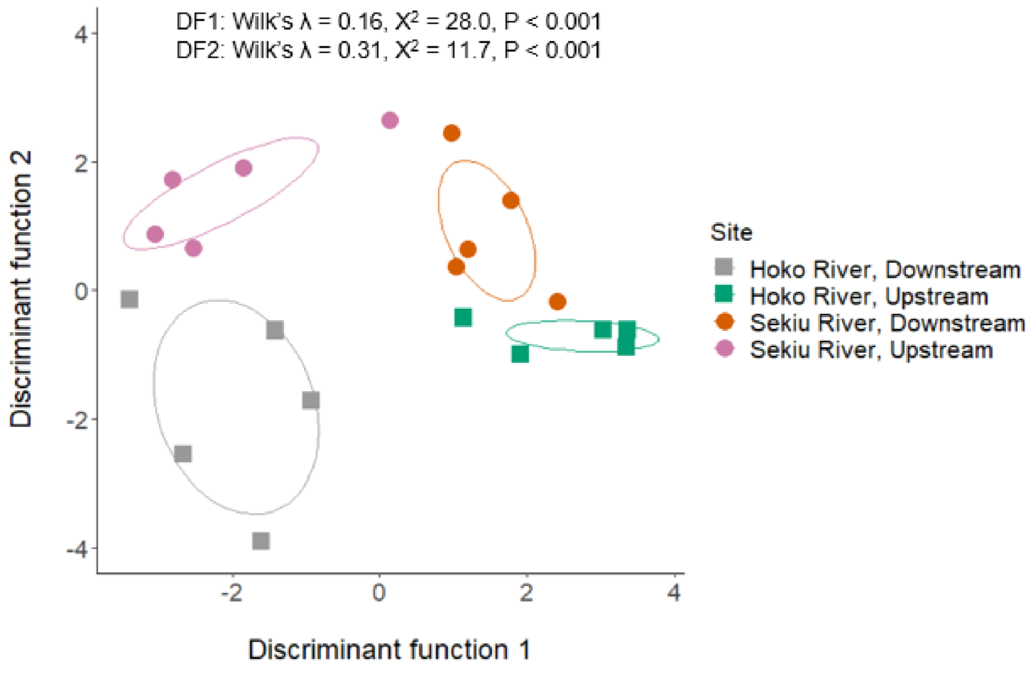

(B)

| Peak # | Chemical ID | DF1 | DF2 |
|---|---|---|---|
| 1 | | -0.39 | 0.10 |
| 2 | | -0.63 | 0.00 |
| 3 | HHDP-Glucose | 0.40 | -0.35 |
| 4 | HHDP-Glucose | -0.46 | 0.45 |
| 5 | galloyl glucose | -0.09 | 0.01 |
| 6 | di-HHDP-glucose (Pedunculagin B) | 0.10 | -0.20 |
| 7 | di-HHDP-glucose (Pedunculagin a) | -0.17 | 0.46 |
| 8 | HHDP-galloyl-glucose (Isostrictinin) | -0.34 | -0.16 |
| 9 | di-galloyl-HHDP-glucose (Tellimagrandin I B) | 0.16 | 0.48 |
| 10 | HHDP-galloyl-glucose (Strictinin) | -0.55 | 0.01 |
| 11 | | 0.10 | 0.09 |
| 12 | di-galloyl-HHDP-glucose (Tellimagrandin I a) | -0.02 | 0.32 |
| 13 | | 0.71 | 0.10 |
| 14 | | -0.42 | 0.10 |
| 15 | di-HHDP-galloyl-glucose B (Casuarictin) | 0.00 | 0.08 |
| 16 | di-HHDP-galloyl-glucose a | -0.61 | 0.05 |
| 17 | | 0.87 | -0.68 |
| 18 | tri-O-galloyl-HHDP-glucose | -0.19 | 0.23 |
| 19 | | -0.34 | 0.49 |
| 20 | | 0.76 | -0.58 |
| 21 | | 0.38 | -1.02 |
| 22 | tri-O-galloyl-HHDP-glucose | 0.05 | 0.36 |
| 23 | HOG | 0.74 | 0.02 |
| 24 | Oregonin | -0.41 | -0.01 |
| 25 | Quercitin-glucuronide | -0.40 | -0.01 |
| 26 | Alnuside A | 0.74 | 0.27 |
| 27 | Alnuside B | -0.28 | 0.98 |
| 28 | | 0.13 | 0.36 |
| 29 | Quercitin-rhamnoside | -0.03 | 0.07 |
| 30 | Methylhirsutanonol | 0.18 | -0.18 |
| 31 | platyphylanolanol-xyloside | 0.01 | 0.62 |
| 32 | Hirsutanone | 0.09 | -0.03 |
| 33 | | -0.50 | -0.10 |
| 34 | novel diarylheptanoid | -0.41 | 0.11 |
| 35 | Alnuside C | 0.22 | -0.24 |

(C)

| Peak | RT (secs) | TIC % Area | M-H (%) | Exact Mass | Diff (ppm) | Diagnostic ions/TIC% first | second | third | fourth | Refs | Annotation Level[6] | Chemical ID, formula (MH-) and (oxidation activity abs/s/mM) |
|---|---|---|---|---|---|---|---|---|---|---|---|---|
| 1 | 115 | 1.7 ± 1.2 | | | | 122.92/100 | 206.88/50 | | | | 4 | |
| 2 | 140 | 5.7 ± 2.6 | | | | 191.05/100 | 267.15/20 | 405.09 | | | 4 | |
| 3 | 200 | 2.0 ± 1.5 | 481.0612 (25) | 481.0624 | -1.18 | 300.9976/100 | | | | 1,2 | 3 | HHDP-Glucose, $C_{20}H_{17}O_{14}$ (3) |
| 4 | 270 | 1.2 ± 0.80 | 481.0632 (25) | 481.0624 | +0.82 | 300.9998/100 | | | | 1,2 | 3 | HHDP-Glucose, $C_{20}H_{17}O_{14}$ (3) |
| 5 | 510 | 0.28 ± 0.21 | 331.0636 (100) | 331.0612 | -3.47 | 169.0119/60 | | | | 1,2 | 3 | galloyl glucose, $C_{13}H_{15}O_{10}$ (0) |
| 6 | 1100 | 7.4 ± 3.3 | 783.0726 (80) | 783.0668 | +3.95 | 300.99/100 | 481.0658 | 301.0015 | 169.0147 | 1,2 | 3 | di-HHDP-glucose, (Pedunculagin β), $C_{34}H_{23}O_{22}$ (5) |
| 7 | 1190 | 9.8 ± 2.5 | 783.0699 (80) | 783.0668 | +1.25 | 300.99/100 | 481.0632 | 301 | 169.0147 | 1,2 | 3 | di-HHDP-glucose (Pedunculagin α), $C_{34}H_{23}O_{22}$ (5) |
| 8 | 1250 | 3.1 ± 0.61 | 633.0739 (25) | 633.0733 | +0.56 | 300.9908/100 | 275.01/20 | | | 1,2 | 3 | HHDP-galloyl-glucose (Isostrictinin), $C_{27}H_{21}O_{18}$ (4.9) |
| 9 | 1295 | 5.0 ± 0.73 | 785.0797 (80) | 785.0843 | -4.60 | 300.99/100 | 275.0213/25 | 829.07/10 | | 1,2 | 3 | di-galloyl-HHDP-glucose(Tellimagrandin Iβ),$C_{34}H_{25}O_{22}$ (3.3) |
| 10 | 1370 | 4.8 ± 1.4 | 633.0726 (25) | 633.0733 | -0.74 | 300.9908/100 | 275.01/20 | | | 1,2 | 3 | HHDP-galloyl-glucose (Strictinin), $C_{27}H_{21}O_{18}$ (3) |
| 11 | 1401 | 1.4 ± 0.51 | 965.0825 (4) | | | 124.0154/100 | 183.029/15 | 300.9998/13 | 783.0692 | | 4 | too small, but major galloyl in + |
| 12 | 1427 | 3.7 ± 1.2 | 785.0882 (86) | 785.0855 | +3.90 | 300.9978/100 | 483.0692/2 | 481.0516/0.3 | 169.01285/12 | 1,2 | 3 | di-galloyl-HHDP-glucose(Tellimagrandin Iα),$C_{34}H_{25}O_{22}$ (3.3) |
| 13 | 1445 | 1.5 ± 0.31 | 965.0748 (2) | | | 300.99/100 | 169.0136/35 | 483.0728/27 | 635.0744/3 | | 4 | |
| 14 | 1467 | 2.0 ± 2.2 | | | | 191.0529/100 | 375.0607/14 | 633.0561/1 | 729.1375/1 | | 4 | derivative of chlorogenic acid |
| 15 | 1491 | 4.9 ± 0.94 | 935.0857 (57) | 935.0737 | -1.8 | 300.99/100 | 785.0676/2 | 633.0622/14 | 169.01/3 | 1,2 | 3 | di-HHDP-galloyl-glucose β (Casuarictin), $C_{41}H_{27}O_{26}$ (4.1) |
| 16 | 1525 | 1.7 ± 0.43 | 935.0789 (21) | 935.0737 | 0.7 | 300.99/100 | 785.0723/2 | 633.0656/10 | 169.0128/4 | 1,2 | 3 | di-HHDP-galloyl-glucose α, $C_{41}H_{27}O_{26}$ (4.1) |
| 17 | 1550 | 1.3 ± 0.24 | | | | 300.99/100 | 169.013/55 | 635.0781/8 | 465.0605/12 | | 4 | |
| 18 | 1570 | 3.1 ± 1.3 | 937.0929 (39) | 937.0952 | -2.35 | 300.99/100 | 767.0562/2 | 465.0658/9 | 169.0141/20 | 1,2 | 3 | tri-O-galloyl-HHDP-glucose (β?), $C_{41}H_{29}O_{26}$ (2) |
| 19 | 1610 | 3.0 ± 1.0 | 1025.07 (2) | | | 300.99/100 | 191/15 | 169/6 | | | 4 | derivative of chlorogenic acid |
| 20 | 1675 | 1.3 ± 0.52 | | | | 300.99/100 | | | | | 4 | |
| 21 | 1705 | 1.7 ± 0.28 | 997.08 (4) | | | 300.99/100 | 617.06/4 | 757.077/8 | 169.01/10 | | 4 | |
| 22 | 1740 | 1.7 ± 0.64 | 937.0961 (17) | 937.0952 | +0.85 | 300.99/100 | 785.0707/2 | 465.0562/3 | 169.0152/12 | 1,2 | 3 | tri-O-galloyl-HHDP-glucose (α?), $C_{41}H_{29}O_{26}$ (2) |
| 23 | 1780 | 1.1 ± 0.89 | 507.1871 (17) | 507.1872 | -0.09 | 205.0874/100 | 327.1249/34 | 121.02/90 | | 3 | 2 | HOG*, $C_{25}H_{31}O_{11}$ |
| 24 | 1840 | 7.8 ± 3.4 | 477.1751 (45) | 477.1766 | -1.52 | 205.0874/60 | 121.02/100 | 327.1249/53 | | 3 | 1 | Oregonin**, $C_{24}H_{29}O_{10}$ |
| 24a | 1915 | | 491.1925 (4) | 491.1922 | +0.23 | 121.0284/100 | 311.12/41 | 205.083/56 | 189.087/38 | 3,4 | 2 | Related to Alnuside B with glucose, $C_{25}H_{30}O_{10}$ |
| 25 | 1960 | 7.6 ± 2.5 | 477.0666 (55) | 477.0675 | -0.86 | 301.0273/100 | 150.9997/24 | 255.0212/4 | 178.9932/8 | 5 | 2 | Quercitin-glucuronide, $C_{21}H_{17}O_{13}$ |
| 25a | 1971 | | 463.0899 (100) | 463.0882 | +1.70 | 271.0226/89 | 300.024/88 | 301.029/51 | 255.0267/35 | 5 | 2 | Quercitin-glucoside, $C_{21}H_{19}O_{12}$ |
| 26 | 2025 | 0.25 ± 0.29 | 461.1858(2) | 461.1817 | +4.19 | 311.1322 | 121.0308/62 | 205.0901/18 | 189.0943/6 | 3 | 2 | Alnuside A, $C_{24}H_{29}O_9$ |
| 27 | 2060 | 1.3 ± 0.65 | 461.1837 (2) | 461.1817 | +1.99 | 311.1309 | 121.0303/62 | 205.0887/18 | | 3 | 2 | Alnuside B, $C_{24}H_{29}O_9$ |
| 28 | 2090 | 1.3 ± 0.65 | 483.012 (15) | | | 300.99 | 299.9957/13 | | | | 4 | |
| 29 | 2170 | 5.7 ± 2.0 | 447.0964 (100) | 447.0933 | +3.12 | 301.0372/61 | 178.9975/3 | 151.0024/3 | 283.0394/6 | 5 | 2 | Quercitin-rhamnoside, $C_{21}H_{19}O_{11}$ |
| 30 | 2230 | 1.5 ± 0.98 | 359.1517 (1) | 359.1500 | +1.69 | 121.028/100 | 205.08/3 | 327.116/2 | | 3 | 2 | Methylhirsutanonol, $C_{20}H_{23}O_6$ |
| 31 | 2250 | 0.63 ± 1.1 | 445.1866 (1) | 445.1868 | -0.19 | 295.1349/100 | 189.0928/45 | 121.0297/10 | 169.0136/1 | 3 | 2 | platyphylanolanol-xyloside, $C_{24}H_{30}O_8$ |
| 32 | 2298 | 1.0 ± 0.68 | 327.1232 (13) | 327.1238 | -0.60 | 121.031/100 | 205.0896/8 | 109.028/11 | | 3 | 2 | Hirsutanone, $C_{19}H_{19}O_5$ |
| 33 | 2310 | 0.26 ± 0.18 | 485 (0) | | | 255.026/100 | 227.03/66 | 121.027/11 | 327.127/6 | | 4 | |
| 34 | 2380 | 2.7 ± 2.0 | 591.2442 (4) | 591.2447 | -0.50 | 205.086/100 | 327.1282/44 | 121.02/34 | | 4 | 3 | novel diarylheptanoid***, $C_{30}H_{39}O_{12}$ |
| 35 | 2405 | 0.45 ± 0.62 | 561.2274(7) | 561.2341 | -1.44 | 205.0897/100 | 121/48 | 327.1274/32 | 109.029/9 | 4 | 2 | Alnuside C, $C_{29}H_{37}O_{11}$ |

References: 1: (Moilanen et al. 2013), 2: (Gu et al. 2013), 3: (Novakavic et al. 2014), 4: (Lv and Shea 2010), 5. (Falcao et al. 2012) 6. (Sumner et al. 2007)

*HOG: 1,7-bis(3,4-dihydroxyphenyl)-5-β-D-xylopyranosyl-3-heptanone

**Oregonin: 1,7-bis(3,4-dihydroxyphenyl)-5-β-D-glycopyranosyl-3-heptanone

*** Novel diarylheptanoid: Alnuside C with glucose replacing xylose, 1,7-bis-(3,4-dihydroxy-phenyl)-5-hydroxy-3-heptanone-5-0-[2-(2-methylbutenoyl)]-β-D-glucopyranoside
