# Supplementary material for: The Origin, Succession, and Predicted Metabolism of Bacterial Communities Associated with Leaf Decomposition
Source: mBio. 2019 Sep 3;10(5):e01703-19. doi: 10.1128/mBio.01703-19 (PMC6722416; doi:10.1128/mBio.01703-19)

ELECTRONIC SUPPLEMENTARY MATERIALS

Fig. S8. As supplementary to Fig. 1 of the main text, here we show the corresponding decomposition measures as non-standardized data points in which decomposition rates within an incubation site have not been adjusted to a $\mu = 0$, s.d. = 1. Categories on the x-axis include from right to left 1.) trees growing immediately upstream of the incubation site and 2.) trees growing further upstream of that incubation site at the 'Away Site' on the same river, both of which are considered 'Home,' and then 3.) trees growing downstream of the incubation site at the 'Away Site' on the same river, 4) trees growing at the upstream site on the paired river, and 5.) trees growing at the downstream site on the paired river, all three of which are considered 'Away.' Note that all points are horizontally jittered to minimize overplotting. Also note that leaf mass remaining can be above 100% because leaves absorb excess water when submerged in rivers. See supplementary methods for details of laboratory experiment that was completed to determine a regression equation for converted submerged leaf weight to fresh leaf weight (fresh weight = 0.941(blotted-dry mass) – 0.00337 (R^2 = 0.983).

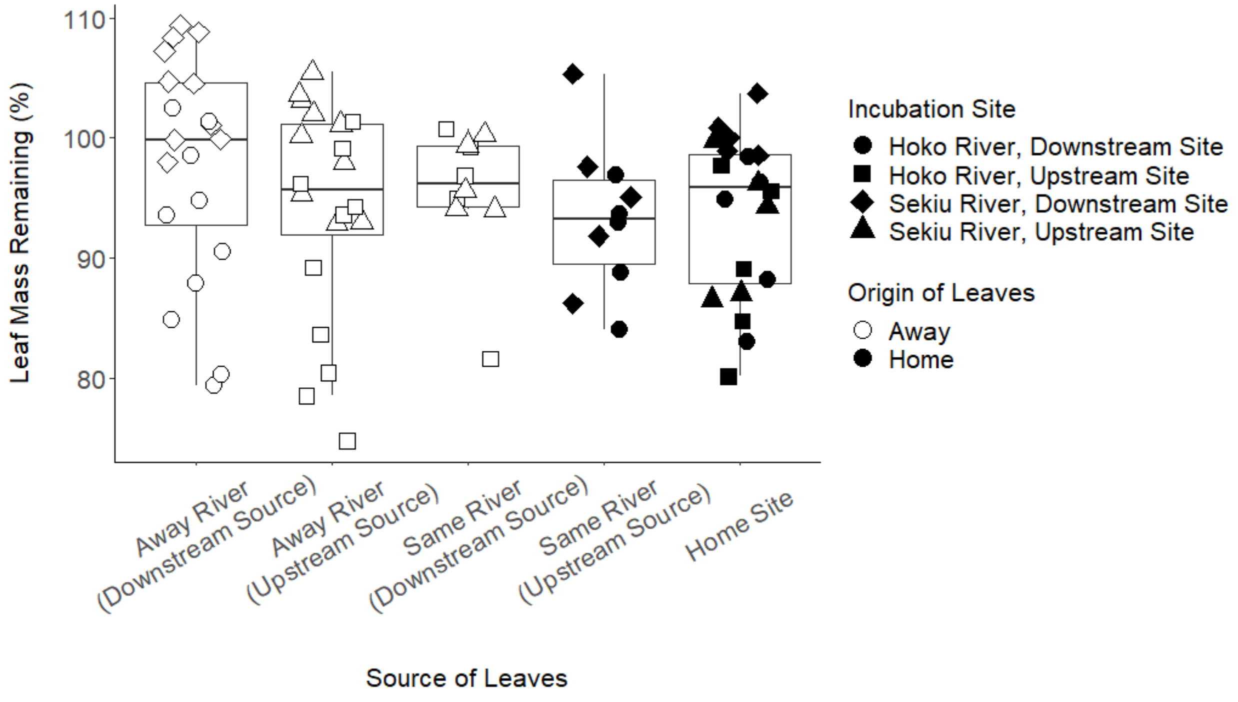

Supplement: FIG S8 [file mBio.01703-19-sf008.pdf]
