# Supplementary material for: The Origin, Succession, and Predicted Metabolism of Bacterial Communities Associated with Leaf Decomposition
Source: mBio. 2019 Sep 3;10(5):e01703-19. doi: 10.1128/mBio.01703-19 (PMC6722416; doi:10.1128/mBio.01703-19)
Supplement: FIG S3 [file mBio.01703-19-sf003.pdf]

# Electronic Supplementary Materials

**Fig. S3**. Community similarity illustrated using the phylogenetic distance metrics, (A) unweighted and (B) weighted UNIFRAC. Communities taxonomically described using 16S rRNA marker gene surveys cluster in principal component space by habitat riparian soil, river water, and leaves either taken directly from riparian red alder trees, or from red alder leaf packs submerged underwater on the streambed. All groups differ significantly from each other all pairwise ANOSIMs $p < 0.01$. (C) Further, we describe the relative abundance of bacterial taxa in summary tables for each environment. Despite containing thousands of rare taxa, environmental samples were dominated by relatively few particularly abundant taxa. Among aquatic leaves, note the decline in Comamonadaceae over time. In contrast to the community inhabiting these leaves, the adjacent water column samples remain largely stable over time. The first column shows the relative abundance in the water column, and the second column show the relative abundance on leaves. Taxa included in this table comprised at least 1% of the bacterial community averaged across all leaf packs samples for the noted time point.

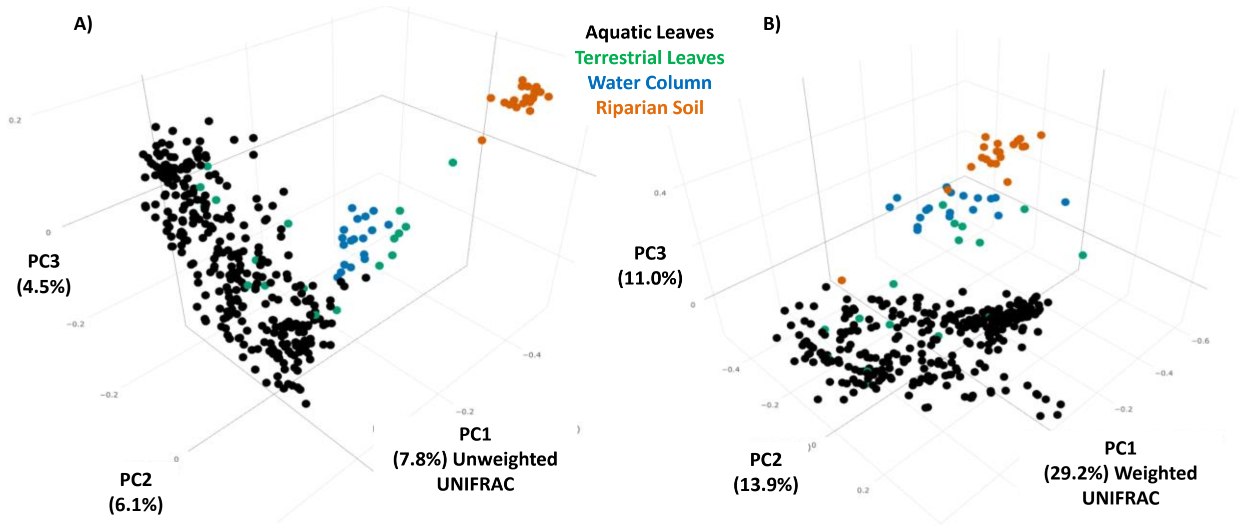

(C)

WATER COLUMN

| Day: 0 | Percent Community Composition of Bacterial Taxa |
|---|---|
| 25.7% | k__Bacteria;p__Actinobacteria;c__Actinobacteria;o__Actinomycetales;f__Microbacteriaceae |
| 20.2% | k__Bacteria;p__Bacteroidetes;c__Flavobacteriia;o__Flavobacteriales;f__Flavobacteriaceae;g__Flavobacterium |
| 15.1% | k__Bacteria;p__Proteobacteria;c__Betaproteobacteria;o__Burkholderiales;f__Comamonadaceae |
| 11.2% | k__Bacteria;p__Bacteroidetes;c__Cytophagia;o__Cytophagales;f__Cytophagaceae |
| 6.5% | k__Bacteria;p__Proteobacteria;c__Betaproteobacteria;o__Burkholderiales;f__Comamonadaceae;Other |
| **Day: 5** | |
| 25.5% | k__Bacteria;p__Actinobacteria;c__Actinobacteria;o__Actinomycetales;f__Microbacteriaceae |
| 15.2% | k__Bacteria;p__Bacteroidetes;c__Flavobacteriia;o__Flavobacteriales;f__Flavobacteriaceae;g__Flavobacterium |
| 14.8% | k__Bacteria;p__Proteobacteria;c__Betaproteobacteria;o__Burkholderiales;f__Comamonadaceae |
| 14.5% | k__Bacteria;p__Bacteroidetes;c__Cytophagia;o__Cytophagales;f__Cytophagaceae |
| 5.4% | k__Bacteria;p__Proteobacteria;c__Betaproteobacteria;o__Burkholderiales;f__Comamonadaceae;Other |
| 5.4% | k__Bacteria;p__Bacteroidetes;c__Flavobacteriia;o__Flavobacteriales;f__Cryomorphaceae;g__Fluviicola |
| **Day: 10** | |
| 28.7% | k__Bacteria;p__Actinobacteria;c__Actinobacteria;o__Actinomycetales;f__Microbacteriaceae |
| 15.7% | k__Bacteria;p__Proteobacteria;c__Betaproteobacteria;o__Burkholderiales;f__Comamonadaceae |
| 14.4% | k__Bacteria;p__Bacteroidetes;c__Flavobacteriia;o__Flavobacteriales;f__Flavobacteriaceae;g__Flavobacterium |
| 9.4% | k__Bacteria;p__Bacteroidetes;c__Cytophagia;o__Cytophagales;f__Cytophagaceae |
| 5.7% | k__Bacteria;p__Proteobacteria;c__Betaproteobacteria;o__Burkholderiales;f__Comamonadaceae;Other |
| **Day: 15** | |
| 30.4% | k__Bacteria;p__Actinobacteria;c__Actinobacteria;o__Actinomycetales;f__Microbacteriaceae |
| 14.7% | k__Bacteria;p__Proteobacteria;c__Betaproteobacteria;o__Burkholderiales;f__Comamonadaceae |
| 11.9% | k__Bacteria;p__Proteobacteria;c__Betaproteobacteria;o__Burkholderiales;f__Comamonadaceae;Other |
| 10.1% | k__Bacteria;p__Bacteroidetes;c__Flavobacteriia;o__Flavobacteriales;f__Flavobacteriaceae;g__Flavobacterium |
| 8.2% | k__Bacteria;p__Bacteroidetes;c__Cytophagia;o__Cytophagales;f__Cytophagaceae |
| **Day: 20** | |
| 38.2% | k__Bacteria;p__Actinobacteria;c__Actinobacteria;o__Actinomycetales;f__Microbacteriaceae |
| 14.5% | k__Bacteria;p__Proteobacteria;c__Betaproteobacteria;o__Burkholderiales;f__Comamonadaceae |
| 9.9% | k__Bacteria;p__Bacteroidetes;c__Flavobacteriia;o__Flavobacteriales;f__Flavobacteriaceae;g__Flavobacterium |
| 7.0% | k__Bacteria;p__Bacteroidetes;c__Cytophagia;o__Cytophagales;f__Cytophagaceae |
| 5.3% | k__Bacteria;p__Proteobacteria;c__Betaproteobacteria;o__Burkholderiales;f__Comamonadaceae;Other |
| 5.0% | k__Bacteria;p__Actinobacteria;c__Actinobacteria;o__Actinomycetales;f__Microbacteriaceae;g__Candidatus Rhodoluna |

SOIL

| | Percent Community Composition of Bacterial Taxa |
|---|---|
| 4.1% | k__Bacteria;p__Proteobacteria;c__Deltaproteobacteria;o__Myxococcales |
| 4.0% | k__Bacteria;p__Proteobacteria;c__Alphaproteobacteria;o__Rhizobiales;f__Hyphomicrobiaceae;g__Rhodoplanes |
| 3.2% | k__Bacteria;p__Proteobacteria;c__Gammaproteobacteria;o__Xanthomonadales;f__Sinobacteraceae |
| 3.1% | k__Bacteria;p__Acidobacteria;c__Acidobacteria-6;o__iii1-15 |
| 3.1% | k__Bacteria;p__Bacteroidetes;c__[Saprospirae];o__[Saprospirales];f__Chitinophagaceae |
| 2.6% | k__Bacteria;p__Proteobacteria;c__Alphaproteobacteria;o__Rhizobiales;f__Bradyrhizobiaceae |
| 2.1% | k__Bacteria;p__Proteobacteria;c__Betaproteobacteria |
| 2.1% | k__Bacteria;p__Acidobacteria;c__Acidobacteriia;o__Acidobacteriales;f__Koribacteraceae |
| 1.9% | k__Bacteria;p__Acidobacteria;c__DA052;o__Ellin6513 |
| 1.9% | k__Bacteria;p__Proteobacteria;c__Alphaproteobacteria;o__Rhodospirillales;f__Rhodospirillaceae |
| 1.8% | k__Bacteria;p__Acidobacteria;c__Solibacteres;o__Solibacterales |
| 1.8% | k__Bacteria;p__Proteobacteria;c__Betaproteobacteria;o__Burkholderiales;f__Comamonadaceae |

TERRESTRIAL LEAVES

| | Percent Community Composition of Bacterial Taxa |
|---|---|
| 12.2% | k__Bacteria;p__Proteobacteria;c__Betaproteobacteria;o__Burkholderiales;f__Comamonadaceae |
| 3.6% | k__Bacteria;p__Proteobacteria;c__Gammaproteobacteria;o__Alteromonadales;f__Alteromonadaceae |
| 3.3% | k__Bacteria;p__Bacteroidetes;c__Bacteroidia;o__Bacteroidales;f__Bacteroidaceae;g__Bacteroides |
| 3.3% | k__Bacteria;p__Bacteroidetes;c__Cytophagia;o__Cytophagales;f__Cytophagaceae;g__Flectobacillus |
| 2.8% | k__Bacteria;p__Proteobacteria;c__Alphaproteobacteria;o__Rhizobiales;f__Rhizobiaceae;g__Agrobacterium |
| 2.7% | k__Bacteria;p__Proteobacteria;c__Alphaproteobacteria;o__Sphingomonadales;f__Sphingomonadaceae;g__Novosphingobium |
| 2.6% | k__Bacteria;p__Proteobacteria;c__Alphaproteobacteria;o__Rhodobacterales;f__Rhodobacteraceae;g__Rhodobacter |
| 2.5% | k__Bacteria;p__Proteobacteria;c__Betaproteobacteria;o__Burkholderiales;f__Comamonadaceae;Other |
| 2.4% | k__Bacteria;p__Tenericutes;c__Mollicutes;o__Acholeplasmatales;f__Acholeplasmataceae;g__Candidatus Phytoplasma |
| 2.1% | k__Bacteria;p__Proteobacteria;c__Alphaproteobacteria;o__Sphingomonadales;f__Sphingomonadaceae |
| 2.0% | k__Bacteria;p__Proteobacteria;c__Deltaproteobacteria;o__Myxococcales |
| 1.9% | k__Bacteria;p__Proteobacteria;c__Alphaproteobacteria;o__Rhodobacterales;f__Rhodobacteraceae |

AQUATIC LEAVES

| Day: 5 | | Percent Community Composition of Bacterial Taxa |
|---|---|---|
| Water Column | Leaves | |
| 20.2% | 54.7% | k__Bacteria;p__Proteobacteria;c__Betaproteobacteria;o__Burkholderiales;f__Comamonadaceae |
| 0.1% | 5.7% | k__Bacteria;p__Proteobacteria;c__Alphaproteobacteria;o__Sphingomonadales;f__Sphingomonadaceae;g__Novosphingobium |
| 0.1% | 3.4% | k__Bacteria;p__Proteobacteria;c__Deltaproteobacteria;o__Myxococcales |
| 0.6% | 3.0% | k__Bacteria;p__Proteobacteria;c__Alphaproteobacteria;o__Rhodobacterales;f__Rhodobacteraceae;g__Rhodobacter |
| 0.05% | 2.1% | k__Bacteria;p__Bacteroidetes;c__Cytophagia;o__Cytophagales;f__Cytophagaceae;g__Flectobacillus |
| 0.02% | 1.8% | k__Bacteria;p__Proteobacteria;c__Alphaproteobacteria;o__Sphingomonadales;f__Sphingomonadaceae |
| 0.05% | 1.6% | k__Bacteria;p__Proteobacteria;c__Betaproteobacteria;o__Burkholderiales;f__Comamonadaceae;g__Leptothrix |
| 0.01% | 1.5% | k__Bacteria;p__Proteobacteria;c__Gammaproteobacteria;o__Pseudomonadales;f__Moraxellaceae |
| 15.2% | 1.4% | k__Bacteria;p__Bacteroidetes;c__Flavobacteriia;o__Flavobacteriales;f__Flavobacteriaceae;g__Flavobacterium |
| 0.04% | 1.3% | k__Bacteria;p__Proteobacteria;c__Alphaproteobacteria;o__Rhizobiales;f__Rhizobiaceae;g__Agrobacterium |
| 0.01% | 1.1% | k__Bacteria;p__Proteobacteria;c__Alphaproteobacteria;o__Rhizobiales |
| **Day: 10** | | |
| 21.4% | 34.1% | k__Bacteria;p__Proteobacteria;c__Betaproteobacteria;o__Burkholderiales;f__Comamonadaceae |
| 0.2% | 14.7% | k__Bacteria;p__Proteobacteria;c__Deltaproteobacteria;o__Myxococcales |
| 0.1% | 7.0% | k__Bacteria;p__Proteobacteria;c__Alphaproteobacteria;o__Sphingomonadales;f__Sphingomonadaceae;g__Novosphingobium |
| 0.7% | 4.2% | k__Bacteria;p__Proteobacteria;c__Alphaproteobacteria;o__Rhodobacterales;f__Rhodobacteraceae;g__Rhodobacter |
| 0.2% | 3.7% | k__Bacteria;p__Proteobacteria;c__Alphaproteobacteria;o__Sphingomonadales;f__Sphingomonadaceae |
| 0.04% | 2.7% | k__Bacteria;p__Bacteroidetes;c__Cytophagia;o__Cytophagales;f__Cytophagaceae;g__Flectobacillus |
| 14.4% | 1.9% | k__Bacteria;p__Bacteroidetes;c__Flavobacteriia;o__Flavobacteriales;f__Flavobacteriaceae;g__Flavobacterium |
| 0.04% | 1.9% | k__Bacteria;p__Proteobacteria;c__Alphaproteobacteria;o__Rhizobiales;f__Rhizobiaceae;g__Agrobacterium |
| 0.2% | 1.5% | k__Bacteria;p__Proteobacteria;c__Betaproteobacteria;o__Burkholderiales;f__Oxalobacteraceae |
| 0.3% | 1.4% | k__Bacteria;p__Proteobacteria;c__Alphaproteobacteria;o__Rhizobiales |
| 0.2% | 1.3% | k__Bacteria;p__Proteobacteria;c__Gammaproteobacteria;o__Enterobacteriales;f__Enterobacteriaceae;Other |
| 0.1% | 1.2% | k__Bacteria;p__Proteobacteria;c__Betaproteobacteria;o__Methylophilales;f__Methylophilaceae;g__Methylotenera |
| 0.3% | 1.1% | k__Bacteria;p__Bacteroidetes;c__Saprospirae;o__Saprospirales;f__Chitinophagaceae |
| 0.5% | 1.0% | k__Bacteria;p__Proteobacteria;c__Alphaproteobacteria;o__Sphingomonadales |
| **Day: 15** | | |
| 26.6% | 25.9% | k__Bacteria;p__Proteobacteria;c__Betaproteobacteria;o__Burkholderiales;f__Comamonadaceae |
| 0.1% | 7.6% | k__Bacteria;p__Proteobacteria;c__Deltaproteobacteria;o__Myxococcales |
| 0.05% | 5.5% | k__Bacteria;p__Bacteroidetes;c__Cytophagia;o__Cytophagales;f__Cytophagaceae;g__Flectobacillus |
| 1.1% | 5.5% | k__Bacteria;p__Proteobacteria;c__Alphaproteobacteria;o__Rhodobacterales;f__Rhodobacteraceae;g__Rhodobacter |
| 0.2% | 5.4% | k__Bacteria;p__Proteobacteria;c__Alphaproteobacteria;o__Sphingomonadales;f__Sphingomonadaceae;g__Novosphingobium |
| 0.2% | 5.0% | k__Bacteria;p__Proteobacteria;c__Alphaproteobacteria;o__Sphingomonadales;f__Sphingomonadaceae |
| 0.03% | 3.8% | k__Bacteria;p__Proteobacteria;c__Alphaproteobacteria;o__Rhizobiales;f__Rhizobiaceae;g__Agrobacterium |
| 10.1% | 3.4% | k__Bacteria;p__Bacteroidetes;c__Flavobacteriia;o__Flavobacteriales;f__Flavobacteriaceae;g__Flavobacterium |
| 0.3% | 2.9% | k__Bacteria;p__Proteobacteria;c__Alphaproteobacteria;o__Rhizobiales |
| 0.3% | 2.1% | k__Bacteria;p__Bacteroidetes;c__Saprospirae;o__Saprospirales;f__Chitinophagaceae |
| 0.01% | 2.0% | k__Bacteria;p__Proteobacteria;c__Alphaproteobacteria;o__Caulobacterales;f__Caulobacteraceae;g__Asticcacaulis |
| 0.1% | 1.9% | k__Bacteria;p__Proteobacteria;c__Betaproteobacteria;o__Methylophilales;f__Methylophilaceae;g__Methylotenera |
| 0.2% | 1.5% | k__Bacteria;p__Proteobacteria;c__Betaproteobacteria;o__Burkholderiales;f__Oxalobacteraceae |
| 0.4% | 1.4% | k__Bacteria;p__Proteobacteria;c__Alphaproteobacteria;o__Sphingomonadales |
| 0.1% | 1.3% | k__Bacteria;p__Proteobacteria;c__Alphaproteobacteria;o__Caulobacterales;f__Caulobacteraceae |
| 0.1% | 1.1% | k__Bacteria;p__Proteobacteria;c__Betaproteobacteria;o__Methylophilales;f__Methylophilaceae |
| **Day: 20** | | |
| 19.8% | 21.8% | k__Bacteria;p__Proteobacteria;c__Betaproteobacteria;o__Burkholderiales;f__Comamonadaceae |
| 9.9% | 6.3% | k__Bacteria;p__Bacteroidetes;c__Flavobacteriia;o__Flavobacteriales;f__Flavobacteriaceae;g__Flavobacterium |
| 0.03% | 5.1% | k__Bacteria;p__Bacteroidetes;c__Cytophagia;o__Cytophagales;f__Cytophagaceae;g__Flectobacillus |
| 0.05% | 4.8% | k__Bacteria;p__Proteobacteria;c__Alphaproteobacteria;o__Rhizobiales;f__Rhizobiaceae;g__Agrobacterium |
| 0.5% | 4.7% | k__Bacteria;p__Proteobacteria;c__Alphaproteobacteria;o__Rhodobacterales;f__Rhodobacteraceae;g__Rhodobacter |
| 0.4% | 4.0% | k__Bacteria;p__Proteobacteria;c__Alphaproteobacteria;o__Rhizobiales |
| 0.2% | 3.8% | k__Bacteria;p__Proteobacteria;c__Alphaproteobacteria;o__Sphingomonadales;f__Sphingomonadaceae |
| 0.1% | 3.7% | k__Bacteria;p__Proteobacteria;c__Alphaproteobacteria;o__Sphingomonadales;f__Sphingomonadaceae;g__Novosphingobium |
| 0.3% | 3.4% | k__Bacteria;p__Bacteroidetes;c__Saprospirae;o__Saprospirales;f__Chitinophagaceae |
| 0.1% | 3.2% | k__Bacteria;p__Proteobacteria;c__Deltaproteobacteria;o__Myxococcales |
| 0.1% | 2.7% | k__Bacteria;p__Proteobacteria;c__Betaproteobacteria;o__Methylophilales;f__Methylophilaceae;g__Methylotenera |
| 0.02% | 2.1% | k__Bacteria;p__Proteobacteria;c__Alphaproteobacteria;o__Caulobacterales;f__Caulobacteraceae;g__Asticcacaulis |
| 0.1% | 1.6% | k__Bacteria;p__Proteobacteria;c__Alphaproteobacteria;o__Caulobacterales;f__Caulobacteraceae |
| 0.1% | 1.6% | k__Bacteria;p__Proteobacteria;c__Betaproteobacteria;o__Burkholderiales;f__Oxalobacteraceae |
| 0.00% | 1.5% | k__Bacteria;p__Bacteroidetes;c__Cytophagia;o__Cytophagales;f__Cytophagaceae;g__Emticicia |
| 0.6% | 1.5% | k__Bacteria;p__Proteobacteria;c__Alphaproteobacteria;o__Sphingomonadales |
| 0.1% | 1.3% | k__Bacteria;p__Proteobacteria;c__Betaproteobacteria;o__Burkholderiales;f__Comamonadaceae;g__Rubrivivax |
| 0.1% | 1.1% | k__Bacteria;p__Proteobacteria;c__Alphaproteobacteria;o__Rhizobiales;f__Phyllobacteriaceae |
| 0.1% | 1.1% | k__Bacteria;p__Proteobacteria;c__Alphaproteobacteria;o__Rhizobiales;f__Hyphomicrobiaceae;g__Devosia |
| 0.05% | 1.0% | k__Bacteria;p__Proteobacteria;c__Betaproteobacteria;o__Methylophilales;f__Methylophilaceae |
