# Supplementary material for: The Origin, Succession, and Predicted Metabolism of Bacterial Communities Associated with Leaf Decomposition
Source: mBio. 2019 Sep 3;10(5):e01703-19. doi: 10.1128/mBio.01703-19 (PMC6722416; doi:10.1128/mBio.01703-19)

Fig. S4. Bacteria inhabiting red alder leaf packs submerged on riverbeds are derived more so from (A) terrestrial leaves (estimated 66.7 ± 0.9 s.e. %) and (B) unknown sources (29.6 ± 0.9 %), while the water column and riparian soil are not shown as they were more minor contributors (2.9 ± 0.1 % and 0.82 ± 0.04 %, respectively) to the bacterial community according to Bayesian SourceTracker models. Proportions of the bacterial community derived from terrestrial leaves varied significantly by days of incubation and leaf origin, however effects were incubation site specific. Panels indicate site of incubation. Note that all points are horizontally jittered to minimize overplotting.

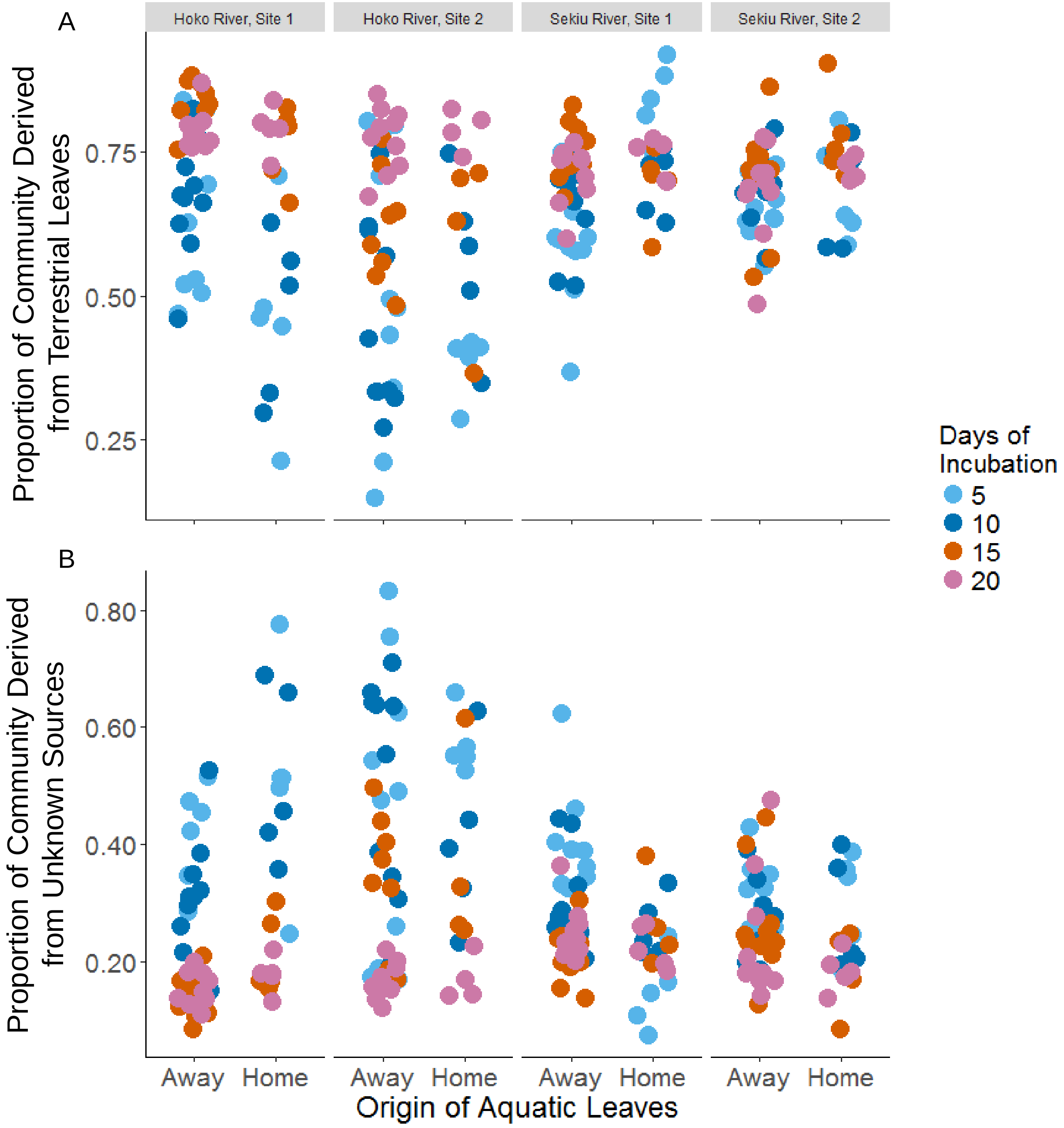

Supplement: FIG S4 [file mBio.01703-19-sf004.pdf]
