# Supplementary material for: The Origin, Succession, and Predicted Metabolism of Bacterial Communities Associated with Leaf Decomposition
Source: mBio. 2019 Sep 3;10(5):e01703-19. doi: 10.1128/mBio.01703-19 (PMC6722416; doi:10.1128/mBio.01703-19)
Supplement: FIG S5 [file mBio.01703-19-sf005.pdf]

Electronic Supplementary Materials

Fig. S5. Certain taxa inhabiting decomposing alder leaf litter varied significantly in relative abundance over time. (A) We characterized each taxon as early, mid, or late successional based on visual inspection of relative abundance plots, as shown below. We defined early-stage taxa as those with highest relative abundance during day 5, mid-stage as those with highest relative abundance during day 10 and 15, and late stage taxa as those with highest relative abundance during day 20. We restricted this analysis to 91 taxa that comprised at least 1% of the community in at least one sample. Instead of averaging across our sample set, this approach included taxa that may comprise a sizable portion of a community, but only in a subset of samples. (B) We also summarize these results in a table where all reported significance values were corrected for multiple comparisons testing using the false discovery rate correction. We also note taxa with significant day x site interactions, as well as taxa with significant day x leaf origin and day x leaf origin x site interactions to identify taxa that may be contributing to accelerated decomposition of local leaves. Only a single taxon in the order Pedosphaerales showed a significant day x leaf origin interactions.

(A)

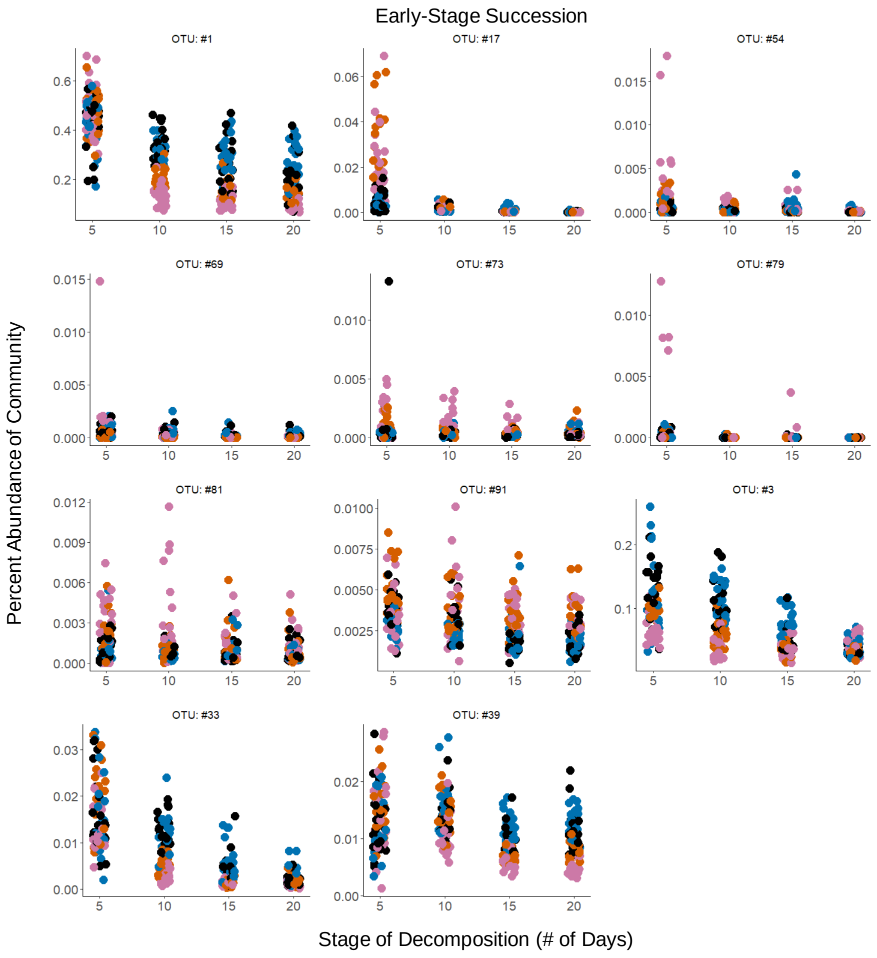

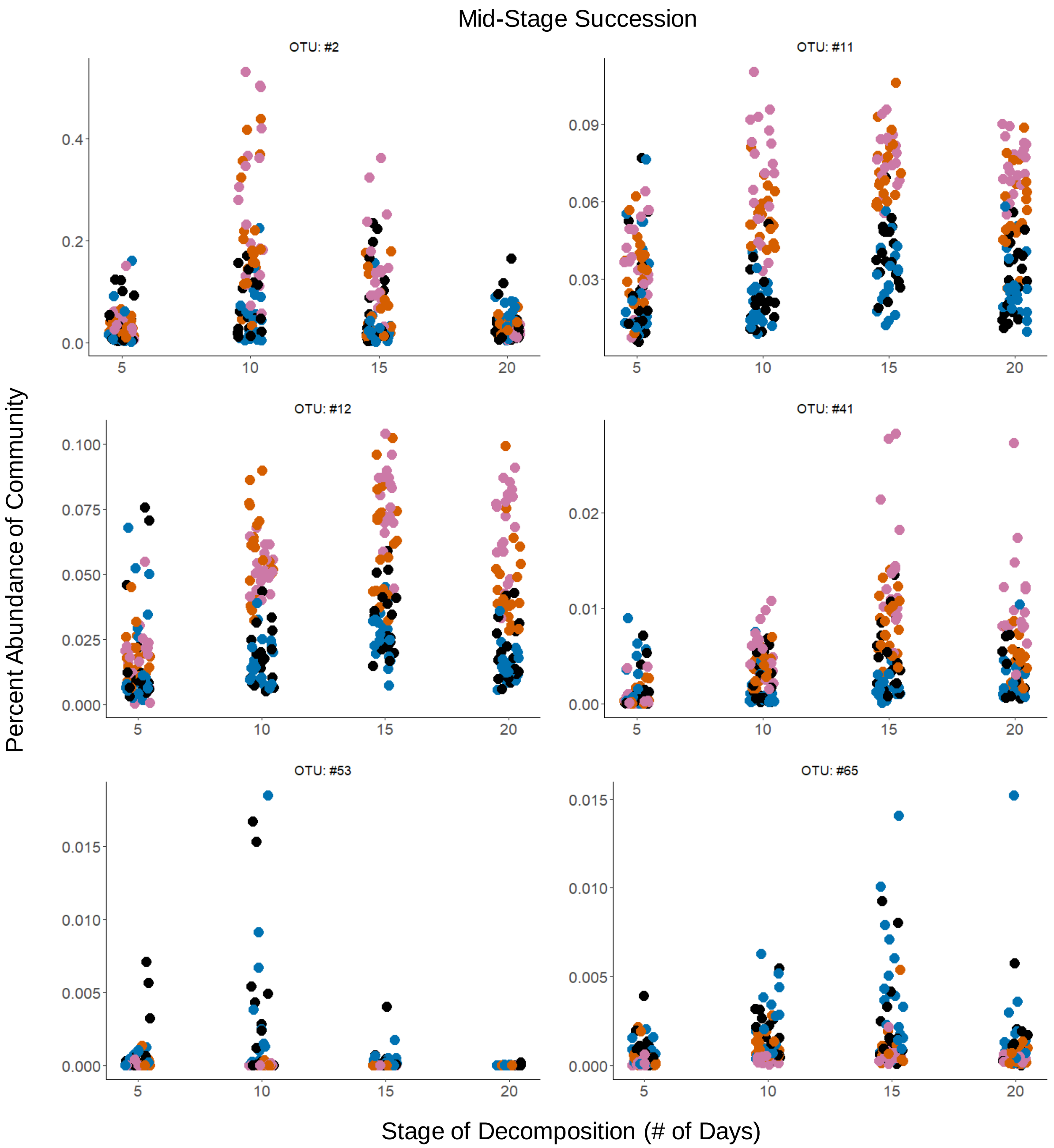
Mid-Stage Succession
OTU: #2
OTU: #11
OTU: #12
OTU: #41
OTU: #53
OTU: #65
0.0
0.2
0.4
0.03
0.06
0.09
0.000
0.025
0.050
0.075
0.100
0.00
0.01
0.02
0.005
0.010
0.015
5
10
15
20
Percent Abundance of Community
Stage of Decomposition (# of Days)

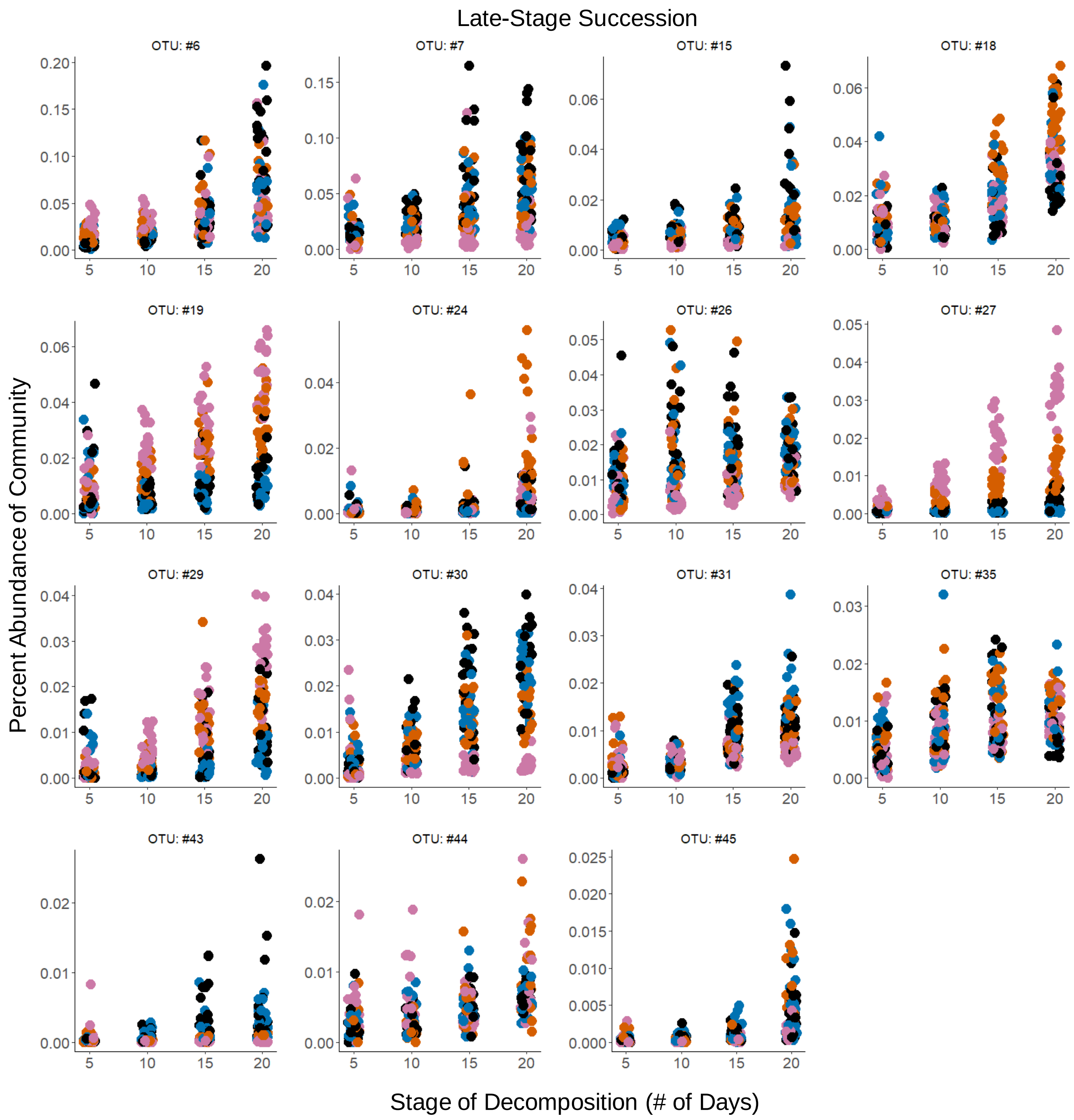
Late-Stage Succession
Percent Abundance of Community
Stage of Decomposition (# of Days)
OTU: #6
OTU: #7
OTU: #15
OTU: #18
OTU: #19
OTU: #24
OTU: #26
OTU: #27
OTU: #29
OTU: #30
OTU: #31
OTU: #35
OTU: #43
OTU: #44
OTU: #45

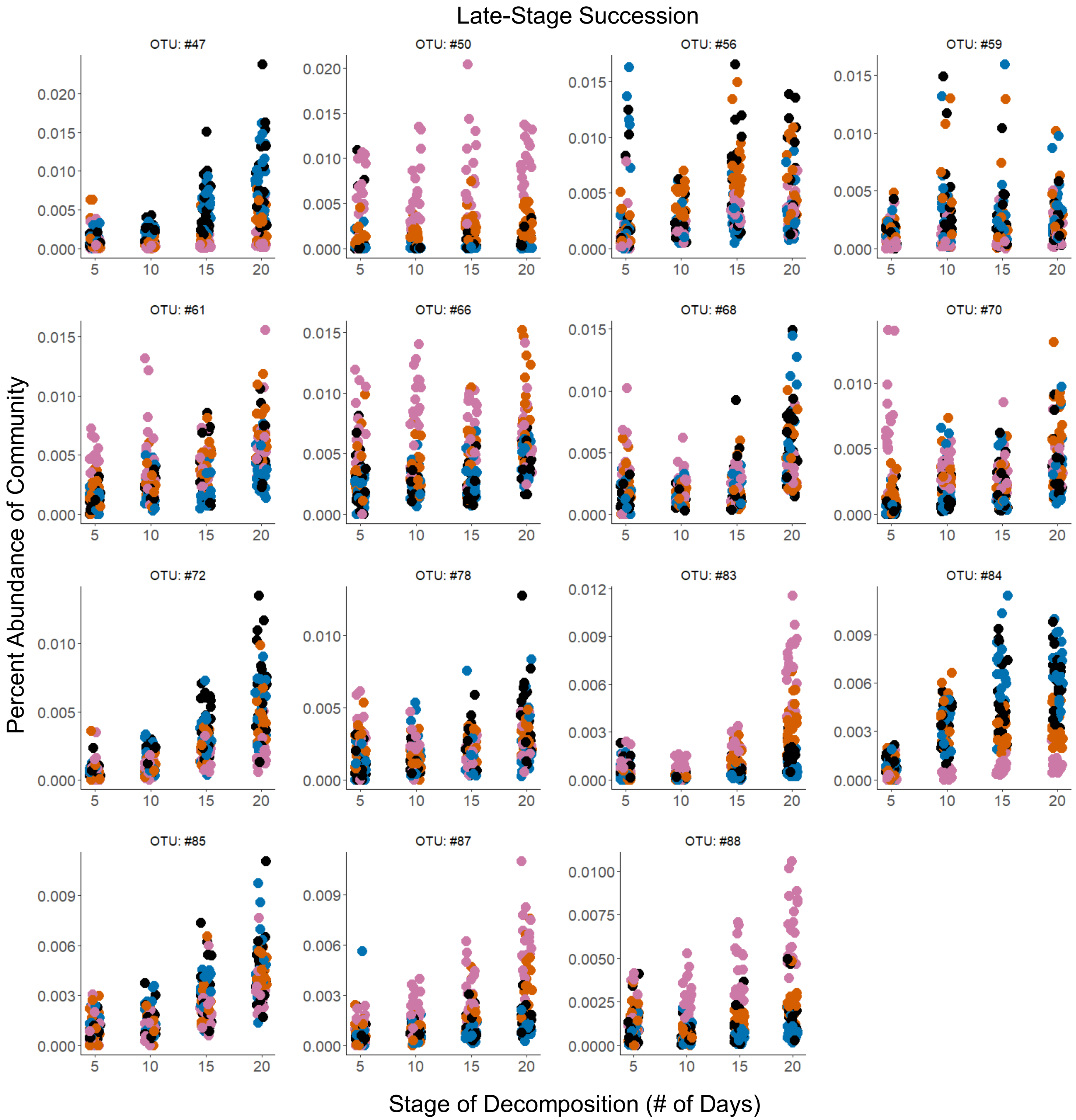
Late-Stage Succession
OTU: #47
OTU: #50
OTU: #56
OTU: #59
OTU: #61
OTU: #66
OTU: #68
OTU: #70
OTU: #72
OTU: #78
OTU: #83
OTU: #84
OTU: #85
OTU: #87
OTU: #88
Percent Abundance of Community
Stage of Decomposition (# of Days)

(B)

Taxa Significantly Characteristic of Early-Stage Succession

OTU	OTU #	$F_{3,295}$	p-value	R^2
k__Bacteria;p__Proteobacteria;c__Betaproteobacteria;o__Burkholderiales;f__Comamonadaceae	1	194.0	1.6E-67	0.52
k__Bacteria;p__Proteobacteria;c__Betaproteobacteria;o__Burkholderiales;f__Comamonadaceae;Other	3	94.9	4.5E-41	0.33
k__Bacteria;p__Proteobacteria;c__Gammaproteobacteria;o__Pseudomonadales;f__Moraxellaceae	17	105.7	1.6E-44	0.35
k__Bacteria;p__Proteobacteria;c__Betaproteobacteria;o__Burkholderiales;f__Comamonadaceae;g__Leptothrix	33	173.6	6.9E-63	0.55
Unassigned;Other;Other;Other;Other;Other	39	43.6	2.1E-21	0.24
k__Bacteria;p__Proteobacteria;c__Gammaproteobacteria;o__Pseudomonadales;f__Moraxellaceae;g__Acinetobacter	54	16.2	7.8E-08	0.11
k__Bacteria;p__Cyanobacteria;c__Oscillatoriophycideae;o__Oscillatoriales;f__Phormidiaceae;g__Phormidium	69	6.1	4.6E-02	0.04
k__Bacteria;p__Verrucomicrobia;c__Pedosphaerae;o__Pedosphaerales;f__R4-41B	73	9.9	2.8E-04	0.07
k__Bacteria;p__Bacteroidetes;c__Bacteroidia;o__Bacteroidales;f__S24-7;g__	79	6.1	4.3E-02	0.04
k__Bacteria;p__Proteobacteria;c__Alphaproteobacteria;o__Sphingomonadales;f__Sphingomonadaceae;g__Zymomonas	81	6.4	2.8E-02	0.04
k__Bacteria;p__Proteobacteria;c__Betaproteobacteria;o__Burkholderiales;f__Comamonadaceae;g__Methylibium	91	13.4	2.8E-06	0.09

Taxa Significantly Characteristic of Mid-Stage Succession

OTU	OTU #	$F_{3,295}$	p-value	R^2
k__Bacteria;p__Proteobacteria;c__Deltaproteobacteria;o__Myxococcales;f__;g__	2	53.5	1.20E-25	0.24
k__Bacteria;p__Proteobacteria;c__Alphaproteobacteria;o__Rhodobacterales;f__Rhodobacteraceae;g__Rhodobacter	11	44.7	7.20E-22	0.13
k__Bacteria;p__Proteobacteria;c__Alphaproteobacteria;o__Sphingomonadales;f__Sphingomonadaceae	12	71.6	7.30E-33	0.21
k__Bacteria;p__Proteobacteria;c__Alphaproteobacteria;o__Sphingomonadales;f__Sphingomonadaceae;g__Sphingobium	41	58.9	6.60E-28	0.23
k__Bacteria;p__Proteobacteria;c__Epsilonproteobacteria;o__Campylobacterales;f__Campylobacteraceae;g__Arcobacter	53	8.2	2.60E-03	0.06
k__Bacteria;p__Proteobacteria;c__Alphaproteobacteria;o__Sphingomonadales;Other;Other	65	9.6	4.00E-04	0.06

Taxa Significantly Characteristic of Late-Stage Succession

OTU	OTU #	$F_{3,295}$	p-value	R^2
k__Bacteria;p__Bacteroidetes;c__Flavobacteriia;o__Flavobacteriales;f__Flavobacteriaceae;g__Flavobacterium	6	64.4	4.30E-30	0.35
k__Bacteria;p__Proteobacteria;c__Alphaproteobacteria;o__Rhizobiales;f__Rhizobiaceae;g__Agrobacterium	7	50.8	1.6E-24	0.26
k__Bacteria;p__Proteobacteria;c__Betaproteobacteria;o__Burkholderiales;f__Comamonadaceae;g__Rubrivivax	15	25.7	7.6E-13	0.16
k__Bacteria;p__Bacteroidetes;c__Saprospirae;o__Saprospirales;f__Chitinophagaceae	18	165.8	4.9E-61	0.53
k__Bacteria;p__Proteobacteria;c__Betaproteobacteria;o__Methylophilales;f__Methylophilaceae;g__Methylotenera	19	96.7	1.2E-41	0.25
k__Bacteria;p__Actinobacteria;c__Actinobacteria;o__Actinomycetales;f__Micromonosporaceae;g__Actinoplanes	24	35.8	8.3E-18	0.19
k__Bacteria;p__Proteobacteria;c__Betaproteobacteria;o__Burkholderiales;f__Oxalobacteraceae	26	10.4	1.4E-04	0.07
k__Bacteria;p__Proteobacteria;c__Alphaproteobacteria;o__Rhizobiales;f__Phyllobacteriaceae	27	170.2	4.5E-62	0.18
k__Bacteria;p__Bacteroidetes;c__Cytophagia;o__Cytophagales;f__Cytophagaceae;g__Emticicia	29	126.4	1.0E-50	0.38
k__Bacteria;p__Proteobacteria;c__Alphaproteobacteria;o__Caulobacterales;f__Caulobacteraceae	30	109.8	8.4E-46	0.34
k__Bacteria;p__Proteobacteria;c__Alphaproteobacteria;o__Rhizobiales;f__Hyphomicrobiaceae;g__Devosia	31	98.1	4.2E-42	0.40
k__Bacteria;p__Proteobacteria;c__Betaproteobacteria;o__Methylophilales;f__Methylophilaceae	35	28.7	2.4E-14	0.20
k__Bacteria;p__Proteobacteria;c__Deltaproteobacteria;o__MIZ46;f__;g__	43	16.2	7.8E-08	0.10
k__Bacteria;p__Verrucomicrobia;c__Verrucomicrobiae;o__Verrucomicrobiales;f__Verrucomicrobiaceae	44	30.1	4.8E-15	0.20
k__Bacteria;p__Proteobacteria;c__Gammaproteobacteria;o__Xanthomonadales;f__Sinobacteraceae;g__Steroidobacter	45	54.9	2.9E-26	0.32
k__Bacteria;p__Proteobacteria;c__Alphaproteobacteria;o__Caulobacterales;f__Caulobacteraceae;g__Caulobacter	47	63.0	1.6E-29	0.24
k__Bacteria;p__Bacteroidetes;c__Cytophagia;o__Cytophagales;f__Cytophagaceae;g__Leadbetterella	50	7.3	9.4E-03	0.02
k__Bacteria;p__Proteobacteria;c__Alphaproteobacteria;o__Sphingomonadales;f__Sphingomonadaceae;Other	56	19.6	1.2E-09	0.12
k__Bacteria;p__Proteobacteria;c__Gammaproteobacteria;o__;f__;g__	59	6.6	2.2E-02	0.05
k__Bacteria;p__Planctomycetes;c__Planctomycetia;o__Pirellulales;f__Pirellulaceae	61	46.1	1.6E-22	0.26
k__Bacteria;p__Bacteroidetes;c__Sphingobacteriia;o__Sphingobacteriales	66	19.8	9.2E-10	0.09
k__Bacteria;p__Bacteroidetes;c__Cytophagia;o__Cytophagales;f__Cytophagaceae	68	64.1	5.7E-30	0.35
k__Bacteria;p__Bacteroidetes;c__Flavobacteriia;o__Flavobacteriales;f__Cryomorphaceae;g__Fluviicola	70	18.0	8.2E-09	0.10
k__Bacteria;p__Proteobacteria;c__Alphaproteobacteria;o__Rhodobacterales;f__Hyphomonadaceae	72	126.8	8.2E-51	0.46
k__Bacteria;p__Bacteroidetes;c__Saprospirae;o__Saprospirales;f__Saprospiraceae	78	15.6	1.7E-07	0.11
k__Bacteria;p__Acidobacteria;c__Chloracidobacteria;o__RB41;f__Ellin6075	83	213.4	1.3E-71	0.36
k__Bacteria;p__Proteobacteria;c__Alphaproteobacteria;o__BD7-3	84	122.9	1.0E-49	0.29
k__Bacteria;p__Armatimonadetes;c__Fimbriimonadia;o__Fimbriimonadales;f__Fimbriimonadaceae;g__Fimbriimonas	85	136.9	1.3E-53	0.54
k__Bacteria;p__Verrucomicrobia;c__partobacteria;o__Chthoniobacterales;f__Chthoniobacteraceae	87	81.1	2.5E-36	0.25
k__Bacteria;p__Proteobacteria;c__Alphaproteobacteria;o__Rhizobiales;f__Hyphomicrobiaceae;g__Hyphomicrobium	88	41.7	1.50E-20	0.11

Successional Patterns Signficant but Variable by Incubation Site

OTU	OTU #	$F_{3,295}$	p-value	R^2
k__Bacteria;p__Bacteroidetes;c__Cytophagia;o__Cytophagales;f__Cytophagaceae;g__Flectobacillus	4	28.5	2.9E-14	0.11
k__Bacteria;p__Proteobacteria;c__Alphaproteobacteria;o__Sphingomonadales;f__Sphingomonadaceae;g__Novosphingobium	5	22.1	5.9E-11	0.14
k__Bacteria;p__Proteobacteria;c__Alphaproteobacteria;o__Rhizobiales	8	139.1	3.3E-54	0.21
k__Bacteria;p__Proteobacteria;c__Alphaproteobacteria;o__Caulobacterales;f__Caulobacteraceae;g__Asticcacaulis	10	32.0	5.9E-16	0.14
k__Bacteria;p__Proteobacteria;c__Gammaproteobacteria;o__Aeromonadales;f__Aeromonadaceae;g__Tolumonas	14	12.5	9.8E-06	0.08
k__Bacteria;p__Proteobacteria;c__Gammaproteobacteria;o__Enterobacteriales;f__Enterobacteriaceae	20	6.6	2.2E-02	0.04
k__Bacteria;p__Spirochaetes;c__Spirochaetes;o__Spirochaetales;f__Spirochaetaceae;g__Spirochaeta	21	24.6	2.8E-12	0.10
k__Bacteria;p__Proteobacteria;c__Alphaproteobacteria;o__Rhizobiales;Other;Other	22	35.6	1.1E-17	0.09
k__Bacteria;p__Proteobacteria;c__Alphaproteobacteria;o__Sphingomonadales	23	36.1	6.3E-18	0.06
k__Bacteria;p__Bacteroidetes;c__Sphingobacteriia;o__Sphingobacteriales;f__Sphingobacteriaceae	28	12.1	1.6E-05	0.07
k__Bacteria;p__Proteobacteria;c__Betaproteobacteria;o__Rhodocyclales;f__Rhodocyclaceae;g__Thauera	32	8.2	2.6E-03	0.05
k__Bacteria;p__Bacteroidetes;c__Saprospirae;o__Saprospirales;f__Chitinophagaceae;g__Chitinophaga	46	21.2	1.6E-10	0.13
k__Bacteria;p__Bacteroidetes;c__Saprospirae;o__Saprospirales;f__Chitinophagaceae;g__Sediminibacterium	49	14.7	5.2E-07	0.05
k__Bacteria;p__Proteobacteria;c__Alphaproteobacteria;o__Rhizobiales;f__Rhizobiaceae;Other	58	10.9	7.1E-05	0.07
k__Bacteria;p__Proteobacteria;c__Betaproteobacteria;o__Burkholderiales;f__Comamonadaceae;g__Limnobacter	77	8.3	2.4E-03	0.05
k__Bacteria;p__Firmicutes;c__Clostridia;o__Clostridiales;f__Veillonellaceae;g__Pelosinus	89	17.4	1.8E-08	0.11
k__Bacteria;p__Proteobacteria;c__Betaproteobacteria;o__Neisseriales;f__Neisseriaceae;Other	90	7.5	6.9E-03	0.04

Significant Day x Site x Leaf Origin Interaction

OTU	OTU #	$F_{3,295}$	p-value	R^2
k__Bacteria;p__Proteobacteria;c__Gammaproteobacteria;o__Legionellales;f__Coxiellaceae;g__Rickettsiella	63	5.1	< 0.001	0.01
Unassigned;Other;Other;Other;Other;Other	39	4.9	< 0.001	0.24
k__Bacteria;p__Proteobacteria;c__Betaproteobacteria;o__Burkholderiales;f__Comamonadaceae;Other	3	4.1	0.0058	0.33
k__Bacteria;p__Proteobacteria;c__Betaproteobacteria;o__Burkholderiales;f__Comamonadaceae;g__Leptothrix	33	3.5	0.039	0.55
k__Bacteria;p__Proteobacteria;c__Gammaproteobacteria;o__Thiotrichales;f__Thiotrichaceae	82	3.3	0.078	0.01
