# Supplementary material for: The Origin, Succession, and Predicted Metabolism of Bacterial Communities Associated with Leaf Decomposition
Source: mBio. 2019 Sep 3;10(5):e01703-19. doi: 10.1128/mBio.01703-19 (PMC6722416; doi:10.1128/mBio.01703-19)
Supplement: FIG S6 [file mBio.01703-19-sf006.pdf]

Fig. S6. Packs of leaves derived from riparian red alder trees growing a distance away from the incubation site tended to harbor greater bacterial diversity than leaf packs consisting of leaves from the immediately local riparian zone. A linear mixed model of Faith's Phylogenetic Diversity against leaf origin was conducted on five categories of leaf origin with an ordered-ANOVA correction r_sP_C to test our apriori hypothesis that leaves in the Home categories would differ from leaves in the Away categories. For illustration, we show this condensed contrast of Home versus Away that includes all five leaf origin categories reduced to two. Alpha diversity metrics are shown here as standardized scores (i.e., z-scores), in which diversity measures within an incubation site and by each day are adjusted to a $\mu = 0$, s.d. = 1, so a y-axis value = 1 indicates 1 s.d. above the mean alpha diversity measurement for that incubation site from that day. This standardization serves to illustrate the relative diversity measures of the Home versus Away leaf communities, however the mixed-effects model was run on the non-standardized data with day as a fixed effect, and incubation site and tree identity as a random effect. Note points are horizontally jittered to minimize overplotting.

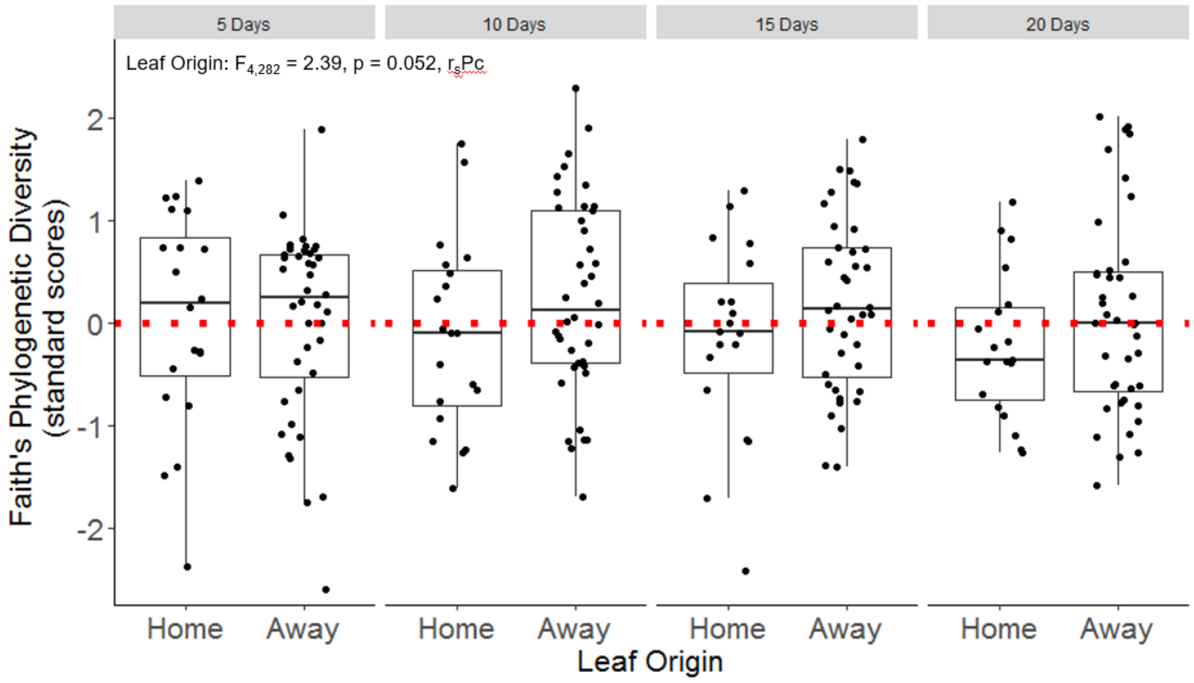

	Df	SS	MS	F	Pr(>F)	r_sP_C
Leaf Origin	4	32527	8132	2.39	0.052	< 0.01
Day	3	12890	4296			
Tree	19	96550	7426			
Incubation Site	3	20377987	6792662			
Residuals	282					
