# Supplementary material for: The Origin, Succession, and Predicted Metabolism of Bacterial Communities Associated with Leaf Decomposition
Source: mBio. 2019 Sep 3;10(5):e01703-19. doi: 10.1128/mBio.01703-19 (PMC6722416; doi:10.1128/mBio.01703-19)
Supplement: FIG S7 [file mBio.01703-19-sf007.pdf]

Fig. S7. (A) Illustration of the subset of pathways in the 'Degradation of Aromatic Compounds' pathway that involve the 12 molecular functional terms that were identified as key differences between the bacterial communities inhabiting Home versus Away leaves using principal component analyses. See the PCA in Fig. 3C and panel (B) for further description of the 12 molecular functional terms. The corresponding table lists all the KEGG Ontology molecular functional terms included in the two Pathways included in our analyses using PICRUSt metagenome functional predictions. The 12 bold terms in the 'Degradation of Aromatic Compounds' pathway were key in distinguishing between bacterial communities inhabiting leaves of different leaf origin, while the 8 bold terms in the 'Metabolism of Starch & Sucrose' pathway are those that we studied further for their involvement in cellulose degradation. Further, (C) we report factor loadings for each of these 12 bolded terms in the 'Degradation of Aromatic Compounds' pathway that were used as variables in a principal component analysis that illustrates the distinct bacterial communities inhabiting Home versus Away leaves. Enzyme commission (EC) numbers provide a nomenclature reference and the Description column gives commonly used names.

(A)

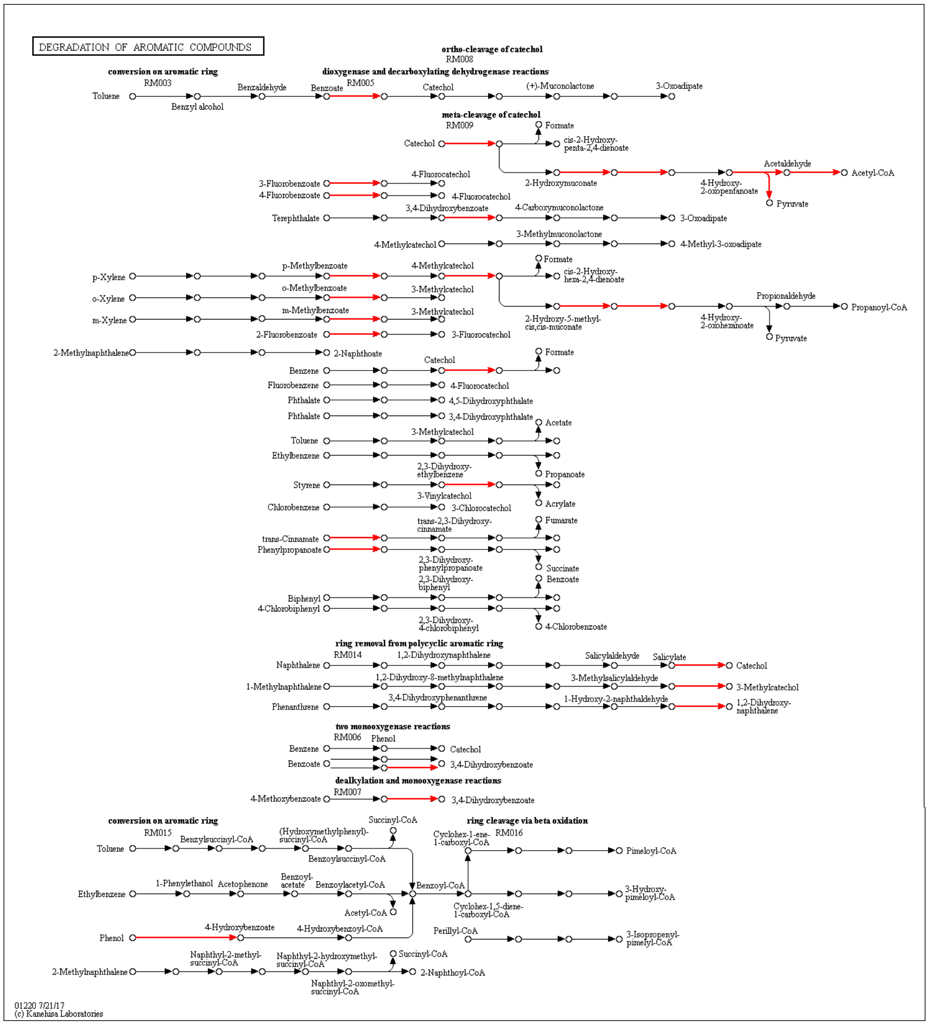

(B)

Degradation of Aromatic Compounds		Metabolism of Starch and Sucrose	
K00001	K06912	K02808	K02438
K00002	K07104	K02809	K01200
K00055	K07535	K02810	K01176
K00121	K07536	K01193	K07405
K00141	K07537	K00696	K05343
K00151	K07538	K07024	K01177
K00152	K07539	K00695	K05992
K00217	K07540	K12047	K01208
**K00446**	K08686	K01187	K01178
K00448	K08689	K12316	K21574
**K00449**	K08690	K12317	K01182
K00455	K10217	K01203	K05988
K00462	K10219	K20811	K00705
**K00480**	K10222	K00692	K16146
**K00481**	K10616	K18775	K16147
K00483	K10617	K01212	K16148
K00484	K10619	K05341	K00691
**K00529**	K10620	K00689	K01838
K01053	K10621	K00690	K02777
K01055	K10622	K00963	K02790
K01607	K10676	K01513	K02791
**K01617**	K10700	K00706	K20107
**K01666**	K10701	K01199	K20108
**K01821**	K10702	K19891	K02749
K01826	K11947	K19892	K02750
K01856	K13953	K19893	K06896
K01857	K13954	K01210	K01232
K02554	K14519	**K01188**	K00701
**K03186**	K14578	**K05349**	K01214
K03268	K14579	**K05350**	K06044
K03379	K14580	K00694	K01236
K03381	K14581	**K01179**	K13057
K03464	K14582	**K19357**	K00697
K04072	K14583	**K01225**	K01087
**K04073**	K14584	**K19668**	K16055
K04105	K14585	**K00702**	K01194
K04107	K14727	K02759	K05342
K04108	K14748	K02760	K02817
K04109	K14749	K02761	K02818
K04112	K14750	K01222	K02819
K04113	K14751	K01223	K01226
K04114		K00978	K01835
K04115		K00975	K15778
K05549		K18447	K15779
K05550		K00703	K00844
**K05708**		K13679	K12407
K05709		K20812	K00845
K05710		K00693	K01084
K05711		K00750	K11809
K05712		K16150	K01810
K05713		K16153	K06859
K05714		K00700	K13810
K05783		K16149	K15916
**K05784**		K00688	K00847
K05921		K01196	

(C)

KEGG	PC1	PC2	EC #	Description
K00529	0.34061	0.03442	1.18.1.3	3-phenylpropionate/trans-cinnamate dioxygenase ferredoxin reductase component
K00449	0.24407	-0.3266	1.13.11.3	protocatechuate 3,4-dioxygenase, beta subunit
K00446	0.32109	0.30797	1.13.11.2	catechol 2,3-dioxygenase
K00481	0.29366	-0.3349	1.14.13.2	p-hydroxybenzoate 3-monooxygenase
K00480	0.23182	-0.4709	1.14.13.1	salicylate hydroxylase
K05708	0.28502	-0.3166	1.14.12.1[illegible]	3-phenylpropionate/trans-cinnamate dioxygenase subunit alpha
K04073	0.30571	0.32959	1.2.1.10	acetaldehyde dehydrogenase
K05784	-0.1348	0.08582	1.18.1	benzoate/toluate 1,2-dioxygenase reductase component
K03186	0.25631	0.33135	2.5.1.129	flavin prenyltransferase
K01617	0.36156	0.12552	4.1.1.77	2-oxo-3-hexenedioate decarboxylase
K01666	0.32134	0.3047	4.1.3.39	4-hydroxy 2-oxovalerate aldolase
K01821	0.298	-0.1697	5.3.2.6	4-oxalocrotonate tautomerase
