# Supplementary material for: The Origin, Succession, and Predicted Metabolism of Bacterial Communities Associated with Leaf Decomposition
Source: mBio. 2019 Sep 3;10(5):e01703-19. doi: 10.1128/mBio.01703-19 (PMC6722416; doi:10.1128/mBio.01703-19)
Supplement: TABLE S1 [file mBio.01703-19-st001.pdf]

ELECTRONIC SUPPLEMENTARY MATERIALS

Table S1. To determine the relative importance of bacterial diversity, predicted functional metabolism, and leaf origin on decomposition rate, we (A) summarized our predictor variables as principal components, and (B) used these principal components in models as composite variables predictive of decomposition rate. (C) We show the analysis of variance table from the best fitting model.

(A)

Null Model: lmer(leaf_mass_remaining~(1|Days_of_Decomposition)+(1|Deployment_Location)+(1|Tree_ID))
PC1 Model: lmer(leaf_mass_remaining~PC1+(1|Days_of_Decomposition)+(1|Deployment_Location)+(1|Tree_ID))
Compared to Null Model: Not significantly different
PC2 Model: lmer(leaf_mass_remaining~PC2+(1|Days_of_Decomposition)+(1|Deployment_Location)+(1|Tree_ID))
Compared to Null Model: LRT X2 = 9.55, p = 0.002
PC3 Model: lmer(leaf_mass_remaining~PC3+(1|Days_of_Decomposition)+(1|Deployment_Location)+(1|Tree_ID))
Compared to Null Model: LRT X2 = 34.3, p < 0.001
PC1 +PC2 + PC3 Model: lmer(leaf_mass_remaining~PC1+PC2+PC3+(1|Days_of_Decomposition)+(1|Deployment_Location)+(1|Tree_ID))
Compared to Null Model: LRT X^2 = 40.0, p < 0.001
Compared to PC3 Model: LRT X^2 = 5.65, p = 0.059

(B)

Element Loadings of Original Variables	PC1	PC2	PC3
Leaf Origin	0.042	**0.59**	**0.81**
Faith's Phylogenetic Diversity	-0.4	**-0.64**	0.46
Predicted Cellulose Degradation	-0.55	**0.5**	-0.36
Predicted Aromatic Degradation	**0.73**	-0.0033	-0.07

(C)

PC1 +PC2 + PC3 Model: ANOVA Table

	DF	SS	MS	F-value
PC1	1	0.00018	0.00018	0.13
PC2	1	0.00018	0.01843	13.48
PC3	1	0.03896	0.03896	28.49
