# Supplementary material for: The Origin, Succession, and Predicted Metabolism of Bacterial Communities Associated with Leaf Decomposition
Source: mBio. 2019 Sep 3;10(5):e01703-19. doi: 10.1128/mBio.01703-19 (PMC6722416; doi:10.1128/mBio.01703-19)
Supplement: TEXT S1 [file mBio.01703-19-s0001.pdf]

ELECTRONIC SUPPLEMENTARY MATERIALS

Data Analysis Methods:

To determine whether leaves from locally-derived trees decomposed more rapidly than leaves sourced from non-local trees, we used ordered-ANOVA to account for our a priori prediction based on our prior studies that Home leaves decompose more quickly than Away leaves at each incubation site (Rice and Gaines 1994, Jackrel and Wootton 2014, Jackrel and Wootton 2015, Jackrel et al. 2016). Specifically, we used our ordered prediction to condense the original five-categories of leaf origin to a main contrast of Home versus Away. The Home group consisted of leaves from 1.) trees growing immediately upstream of the incubation site and 2.) trees growing further upstream of that incubation site at the 'Away Site' on the same river. Our Away group consisted of leaves from 3.) trees growing downstream of the incubation site at the 'Away Site' on the same river, 4.) trees growing at the upstream site on the paired river, and 5.) trees growing at the downstream site on the paired river. As aquatic leaf pack experiments result in initial and final leaf masses containing different moisture levels due to underwater leaf submergence, we previously performed experiments to determine the most accurate means of measuring grams of leaf mass loss rather than changes in water content (Jackrel and Wootton 2014). Leaf pack studies frequently use dried leaf litter from autumnal leaf fall in which leaves can be dried to a constant, precise initial mass before deployment. We aimed to find an accurate method appropriate for fresh, green leaves. Using dry weight is undesirable because drying leaves to obtain initial leaf mass fundamentally changes properties of fresh leaves in unnatural ways prior to experimental deployment. Therefore, to find an alternative method appropriate for fresh leaves, we collected 12 leaves from 19 red alder trees. We weighed groups of six leaves per tree at three stages: (1) freshly picked green leaves, (2) submerged in water for 17 days, and then blotted dry with paper towels, (3) oven-dried to a constant mass. The other six leaves per tree were oven-dried before and after submerging in water to estimate mass loss due to handling, leaching, and measurement error. The strongest correlation occurred between pre- and post-oven-dried leaves ($R^2 = 0.991$), which indicates minimal mass loss due to handling, measurement error, and/or leaching. However, the correlation between freshly picked leaf mass and the mass of leaves blotted dry with paper towels was nearly as strong ($R^2 = 0.983$). In contrast, the correlation between freshly picked leaf mass and oven dried leaf mass was appreciably weaker ($R^2 = 0.875$). Therefore, we used the relationship from the analysis of blotted dry leaves to back-calculate fresh mass of leaves following incubation in the rivers: fresh mass = 0.941(blotted-dry mass) – 0.00337 ($R^2 = 0.98$) (Jackrel and Wootton 2014). We also used an arcsine square root transformation on our decomposition rate data to meet assumptions of normality and homogeneity of variances. Our linear mixed effects model used leaf origin as a fixed effect and tree ID and incubation site as random effects. For the purposes of graphically representing our model results, we then converted percentage leaf mass lost data to z-scores that were standardized by incubation site. In practice, either using standard scores as our dependent variable or including incubation site as a fixed effect in the model yielded similar statistical results regarding our leaf origin treatment.

We analyzed our bacterial sequencing data using the QIIME pipeline (Caporaso et al. 2010). We classified operational taxonomic units (OTUs) from Illumina reads at the 97% similarity level using open reference-based clustering with uclust. We assigned a taxonomy using the RDP taxonomic assignment comparing the OTUs sequences against the Greengenes

database, version 13_8. Although the Greengenes database is smaller and less frequently updated than other frequently used databases, such as SILVA, Greengenes was preferable for our study because it is the only database that is compatible with the functional annotation tool PICRUSt, and is also the only database that provides in-depth taxonomic classification to the species level. We first determined which taxa varied over the course of succession from our leaf pack samples by asking whether collection day significantly predicted taxon relative abundance using analysis of variance with a multiple comparisons, false discovery rate correction (Benjamini and Hochberg 1995). Our model included three categorical variables (day, incubation site, leaf origin) and their interactions. We restricted this analysis to 91 OTUs that comprised at least 1% of the community in at least one of our samples, rather than averaging across our sample set, in order to include taxa that may comprise a sizable portion of the community in only a subset of samples. We identified taxa exhibiting consistent trends in relative population size across incubation sites as those taxa that either did not show a significant interaction between day and incubation site, or that showed the same directional trend for each site as determined visually with interaction plots. For those taxa showing consistent trends across incubation sites, we further categorized them as early, mid, or late successional taxa, defined as relative abundance that peaked on day 5, day 10 or 15, or day 20, respectively. We then highlighted taxa where successional patterns were contingent on leaf origin by noting those taxa with a significant interaction term between day and leaf origin. We also examined successional changes through time with principal coordinates analyses using the non-phylogenetically based Bray-Curtis distance metric, as well as the relative abundance-weighted and unweighted phylogenetically-based community distance metric, UNIFRAC (QIIME: principal_coordinates.py). We highlighted the top ten most abundant taxa that changed markedly across this successional gradient using biplot analyses that were superimposed in principal coordinate space (QIIME: make_emperor.py). We then measured changes in alpha diversity by day and by leaf origin using total OTU Number and Faith's Phylogenetic Diversity (QIIME: core_diversity_analyses). We used a mixed-effects linear regression model with our day treatment as a categorical fixed effect and incubation site and tree ID as random effects. We verified that the resulting model met assumptions of normality and homogeneity of variances. We then tested our main contrast that communities that inhabit Home versus Away leaves differed in alpha diversity using a mixed-effects linear regression models with leaf origin and day as our fixed effects and tree ID and incubation site as random effects. Our leaf origin treatment included all five categories as described above, and so we again incorporated our ordered a priori hypothesis that our Home groups would significantly differ in alpha diversity from our Away groups (Rice and Gaines 1994).

We predicted where the bacterial communities inhabiting our leaf packs originated using Bayesian SourceTracker models (Knights et al. 2011). We generated four site-specific models using a uniform prior by setting equal Dirichlet hyperparameters for our known source environments, including the riparian soil, terrestrial leaves, and the water column, and our unknown source environment (i.e., alpha1 = alpha2 = 0.001). Models for each incubation site included only the subset of riparian soil, water column, and leaf pack samples that were collected from or deployed at that site. The complete set of terrestrial leaf samples was used in each model because leaves from each study tree were deployed at each site. In addition to these four site-specific models, we also further subdivided the dataset to generate tree-specific models at each site in which for the leaf packs from a single tree we only consider the terrestrial leaves from that

tree as a potential source. We report only our four site-specific models because tree-specific models indicated similar proportional contributions of each source.

We next tested the effect of both succession (i.e. day) and our leaf origin treatment on the predicted gene content associated with different metabolic pathways of bacterial communities. We predicted metagenome functional content from our 16S survey data using the PICRUSt software package (Phylogenetic Investigation of Communities by Reconstruction of Unobserved States) (Langille et al. 2012). This pipeline uses ancestral-state reconstruction to predict the presence of gene families and then multiplies predicted functions by OTU relative abundances. To verify the accuracy of our functional predictions, we calculated Nearest Sequences Taxon Index for each sample. This is a relative-abundance weighted index that sums the phylogenetic distances for each OTU in a sample to the nearest relative with a sequenced genome and weights these OTU-specific measures by the frequency of the OTU in a sample (Langille et al. 2012). To identify specific molecular functions of interest we use the Kyoto Encyclopedia of Genes and Genomes (KEGG) Database to identify which specific molecular-level functional terms or KEGG terms are involved in certain functional pathways that we hypothesized were important for leaf degradation. We first tested whether bacterial communities differed in their functional capacity to degrade plant secondary metabolites using the KEGG Pathway: 'Degradation of Aromatic Compounds.' This pathway includes degradation reactions that we have inferred would be involved in the metabolism of two major classes of secondary metabolites in red alder, the polyphenol-based ellagitannins and the diarylheptanoids For example, this pathway includes reactions for the degradation of catechol, benzoates, trans cinnamate, and phenolic acids, which are all subunits of alder secondary metabolites.

We next derived a more general metric of the capacity of bacterial communities to degrade plant material. We used the 'Starch & Sucrose Metabolism,' pathway that includes enzymes involved in the degradation of cellulose, including endogluconase and cellulose 1,4-β-cellobiosidase. All KEGG Ontology Terms included in these two pathways at the time of download from < http://www.genome.jp/kegg/pathway.html> in October of 2017 are listed in Table S4. Within the Starch & Sucrose Metabolism pathway we also identified 8 KEGG Terms specifically involved in cellulose degradation that we refer to as the 'Degradation of Cellulose' pathway. For each pathway, we summed across all KEGG terms included in the pathway that were represented in our dataset to obtain a total measure of pathway function per sample. We refer to these new summary variables as the 'summed capacity' of bacterial communities to degrade aromatic compounds, the summed capacity to metabolize starch and sucrose, and the summed capacity to degrade cellulose.

We note that our dataset precludes us from testing whether variation in leaf chemistry drives variation in predicted gene content associated with different metabolic pathways. We have demonstrated that the secondary chemistry of alders can vary over short time scales in response to herbivore stress and/or nutrient availability (Jackrel and Morton, 2018). Therefore, although we have reported leaf chemistry data on the same trees used in the present study in Jackrel et al. 2016, it would be inaccurate to make inferences between bacteria that inhabited leaves in Summer 2013 with chemistry data analyzed from leaves collected in Summer 2012. Instead, we use our prior analyses to determine which metabolic pathways align best with the classes of secondary metabolites that predominate in alder trees growing at our study sites.

We tested whether functional capacities changed through succession (i.e., day) and by leaf origin. First, we tested for changes though succession with simple linear models of day as our continuous predictor variable and the summed function as our response variable. We also

tested whether individual KEGG Terms within the Degradation of Cellulose pathway changed significantly through succession using the same model structure, and a false-discovery rate correction for multiple comparisons. Second, we tested the effect of leaf origin on capacity to degrade aromatic compounds during day 5, because this was when this function was generally greatest. Similarly, we tested the effect of leaf origin on the capacity to metabolize starch and sucrose, as well as more specifically on the capacity to degrade cellulose, during day 20 when these functions were generally greatest. For these analyses we only used leaves for our categories that represented the strongest contrast and had the greatest sample sizes: leaves incubating at the Home Site on the Home River, versus leaves incubating at the Away River. We tested whether these same Home versus Away groups differed in the capacity of their bacterial communities to degrade aromatic compounds using a two-way ANOVA with treatment groups of leaf origin and Incubation Site. Because this result was significant, we then proceeded to run similar analyses testing whether leaf origin differed for each individual KEGG Ontology term in the Degradation of Aromatic Compounds pathway. Terms that were significant after false discovery rate correction were then used as variables in a principal component analyses to examine the distinct functional capacities of bacterial communities inhabiting leaves of Home versus Away origin.

Lastly, we tested whether the composition of the leaf bacterial community alone could predict the rate of leaf decomposition. We then asked whether adding these data describing the leaf bacterial community improved our original model of leaf decomposition that used leaf origin as the sole predictor variable. Our null model included random effects of day, incubation site, and tree ID. We used percentage leaf mass lost as our response variable and a mix of the following predictor variables: Faith's Phylogenetic Diversity as our measure of alpha diversity, summed capacity to degrade aromatic compounds, summed capacity to degrade cellulose, and our 5-category leaf origin treatment. We selected best fitting models by comparing AIC scores. We report marginal R^2 values for the best fitting mixed-effects models to describe variance explained by the fixed factors (Nakagawa and Schielzeth 2013). For the best fitting model, we report the F-statistic for each main effect, as well as the numerator and denominator degrees of freedom using the ANOVA function in R (R Core Team, 2017).

References:

Benjamini Y, Hochberg, Y. 1995. Controlling the false discovery rate: A practical and powerful approach to multiple testing. Journal of the Royal Statistical Society. Series B Methodological 57:289-300.

Jackrel SL, Morton, TC. 2018. Inducible phenotypic plasticity in plants regulates aquatic ecosystem functioning. Oecologia 186: 895-906.

Lundberg DS, Yourstone S, Mieczkowski P, Jones CD, Dang, JL. 2013. Practical innovations for high-throughput amplicon sequencing. Nature Methods 10:999-1002.

R Core Tream. 2017. R: A language and environment for statistical computing. R Foundation for Statistical Computing, Vienna, Australia. https://www.R-project.org/.

Rice WR, Gaines SD. 1994. The ordered-heterogeneity family of tests. Biometrics 50:746-752.

Nakagawa S, Schielzeth H. 2013. A general and simple method for obtaining R^2 from generalized linear mixed-effects models. Methods in Ecology and Evolution 4:133-142.
